# Targeting Oxidative Stress in Carcinogenesis: Oleanolic Acid and Its Molecular Pathways

**DOI:** 10.3390/antiox15010067

**Published:** 2026-01-04

**Authors:** Andrzej Günther, Maciej Kulawik, Szymon Sip, Przemysław Zalewski, Donata Jarmołowska-Jurczyszyn, Przemysław Stawicki, Barbara Bednarczyk-Cwynar

**Affiliations:** 1 Department of Organic Chemistry, Faculty of Pharmacy, Poznan University of Medical Sciences, Collegium Pharmaceuticum 2, (CP.2), Rokietnicka Str. 3, 60-806 Poznan, Poland; andrzej.gunther@me.pl; 2 Department of Pharmacognosy and Biomaterials, Faculty of Pharmacy, Poznan University of Medical Sciences, Collegium Pharmaceuticum 1 (CP.1), Rokietnicka Str. 3, 60-806 Poznan, Poland; maciej.kulawik@student.ump.edu.pl (M.K.); szymonsip@ump.edu.pl (S.S.); pzalewski@ump.edu.pl (P.Z.); 3 Doctoral School, Poznan University of Medical Sciences, Bukowska 70, 60-812 Poznan, Poland; 4 Department of Clinical Pathology, Faculty of Medical Sciences, Poznan University of Medical Sciences, Przybyszewskiego Str. 49, 60-355 Poznan, Poland; donata.jarmolowska-jurczyszyn@ump.edu.pl; 5 Department of Rehabilitation and Physiotherapy, Faculty of Medical Sciences, Poznan University of Medical Sciences, 28 Czerwca 1956 Str., No 135/147, 60-545 Poznań, Poland; pstawicki@ump.edu.pl; 6 Center of Innovative Pharmaceutical Technology (CITF), Rokietnicka Str. 3, 60-806 Poznan, Poland

**Keywords:** triterpenes, oleanolic acid, anticancer activity of triterpenes, anticancer activity of oleanolic acid, antioxidant activity, antioxidant activity of triterpenes, antioxidant activity of oleanolic acid

## Abstract

This narrative review aims to systematize the current knowledge on the dual role of reactive oxygen species and reactive nitrogen species in cancer processes, from their physiological function in redox signaling to their pathological impact in oxidative distress. The mechanisms of biomolecule damage, particularly DNA, and deregulation of signaling pathways induced by excessive ROS/RNS concentrations, which promote neoplastic transformation, are presented. The importance of diet and endogenous antioxidants in cancer prevention is also discussed, emphasizing the role of natural antioxidants in prevention and adjunctive therapy. In this context, oleanolic acid emerges as a promising compound with dual action modulating oxidative stress, capable of balancing cellular redox responses. We discuss the most important antioxidant mechanisms of oleanolic acid, the interconnection of oxidative stress with carcinogenesis-related pathways, anticancer mechanisms mediated by oxidative stress modulation, and structural modifications and modern application techniques that improve its bioavailability, as well as future perspectives on oleanolic acid research in the context of its antioxidant and anticancer activity. Overall, available experimental and preclinical data indicate that oleanolic acid, through pleiotropic modulation of oxidative stress and signaling networks, holds promise as an adjuvant agent in cancer prevention and therapy.

## 1. Introduction

**Figure 1:**
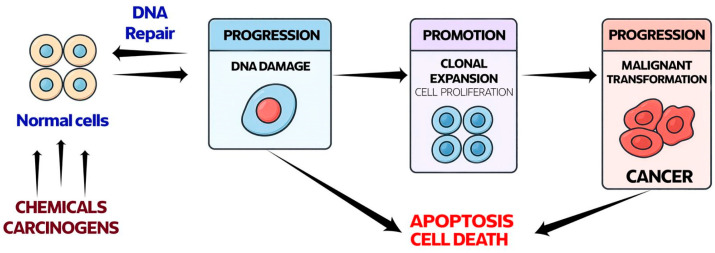
Chemical carcinogenesis stages induced by chemicals.

Carcinogenesis is a multistep process in which normal cells acquire malignant features, including uncontrolled proliferation, evasion of cell-cycle regulation, resistance to apoptosis, and the capacity for tissue invasion and metastasis. It is driven by genetic, epigenetic, and environmental factors and represents the second leading cause of death worldwide, following cardiovascular diseases [1]. Among the key contributors to this process is oxidative stress, resulting from the detrimental activity of free radicals [2]. A diagram of free radical-induced carcinogenesis is shown in Figure 1.

Oxidative stress is defined as an imbalance between the production of reactive oxygen and nitrogen species (ROS/RNS) and the capacity of enzymatic and non-enzymatic antioxidant systems to neutralize them [2]. Key enzymatic antioxidants include superoxide dismutase, glutathione peroxidase, glutathione reductase, and catalase, while major non-enzymatic antioxidants comprise glutathione, vitamins C, E, and A, selenium compounds, and various natural products [2,3]. Oxidative stress may arise from excessive ROS/RNS generation—such as during chronic inflammation, ultraviolet (UV) exposure, toxin contact, or mitochondrial dysfunction—or from impaired antioxidant defenses [2].

Free radicals, though vital for normal physiological functions such as signaling, gene regulation, and immune defense, can become harmful when produced in excess [4]. Their accumulation overwhelms the endogenous antioxidant system, resulting in oxidative stress. This state leads to damage of key biomolecules, e.g., deoxyribonucleic acid (DNA) mutations, lipid peroxidation that disrupts membrane integrity, and protein oxidation that impairs enzymatic and structural functions. Consequently, oxidative stress contributes to cellular dysfunction, accelerated aging, and the development of numerous diseases, including neurodegenerative, cardiovascular, and metabolic disorders and cancer [3].

Numerous studies have demonstrated that oxidative stress contributes to the initiation and progression of a wide range of malignancies, including cancers of the liver, lung, stomach, brain, pancreas, ovary, bladder, breast, cervix, prostate, and oral cavity, as well as melanoma, leukemia, and lymphoma [3]. Importantly, both the incidence of cancer and the associated mortality are showing a steady upward trend, which poses a significant challenge to modern medicine and public health [5].

For this reason, the search for novel and effective pharmacologically active compounds capable of exerting dual activity—both as anticancer agents and as antioxidants—remains an area of great importance in modern biomedical research. Particular attention has been directed toward naturally derived compounds, which are increasingly recognized as promising candidates due to their broad spectrum of biological activities and relatively favorable safety profiles [6].

Among these natural compounds, triterpenes represent a structurally diverse class of secondary plant metabolites with considerable pharmacological potential [7]. Extensive research has demonstrated that triterpenes exhibit a wide range of biological properties, including immunomodulatory effects [8], anti-inflammatory activity [9], antiviral functions [10], and significant anticancer properties [11]. These effects are mediated through their ability to modulate key molecular pathways associated with inflammation, oxidative stress, immune regulation, and carcinogenesis.

Importantly, certain triterpenes have also been shown to possess potent antioxidant properties [12,13], enabling them to mitigate oxidative stress by scavenging free radicals, enhancing endogenous antioxidant defenses, and regulating redox-sensitive signaling cascades. This antioxidant activity, combined with their cytotoxic and pro-apoptotic effects on cancer cells, underscores the multidirectional therapeutic potential of triterpenes, making them highly attractive candidates for further investigation in the context of both cancer prevention and therapy.

Oleanolic acid (OA), an oleanane-type pentacyclic triterpene found in more than 1600 edible and medicinal plant species [14], has attracted considerable scientific interest. Extensive studies have confirmed its broad biological activity [15], with anticancer and antioxidant effects being particularly significant. These activities involve modulation of oxidative stress, regulation of apoptosis, and inhibition of chronic inflammation. Its natural abundance, favorable safety profile, and the ease of generating derivatives with improved properties make OA a promising candidate for targeting oxidative stress in carcinogenesis. Consequently, OA serves not only as a potential preventive and therapeutic agent in oncology but also as a valuable scaffold for designing multifunctional compounds with enhanced pharmacological profiles [15].

In this manuscript, we provide a comprehensive overview and systematization of current knowledge regarding the dual role of reactive oxygen species and reactive nitrogen species in carcinogenesis. Particular emphasis is placed on the molecular mechanisms through which excessive concentrations of ROS and RNS contribute to cellular damage, including oxidative modifications of DNA, proteins, and lipids, as well as on the deregulation of critical signaling pathways that collectively promote genomic instability and neoplastic transformation.

The review further discusses the principal antioxidant mechanisms attributed to OA, including its capacity to regulate key transcription factors and signaling pathways implicated in oxidative stress and inflammation. We also examine the complex interconnections between oxidative stress and carcinogenic signaling cascades, with a focus on how OA-mediated modulation of redox balance can influence tumor initiation, progression, and therapy resistance. In addition, we provide an overview of the anticancer mechanisms of OA that are mediated through its redox-modulating properties, such as the induction of apoptosis, inhibition of proliferation, and regulation of angiogenesis and metastasis.

Attention is also devoted to structural modifications of OA designed to enhance its solubility and bioavailability, as well as to novel formulation strategies, including nanoparticle-based delivery systems, which hold great promise for overcoming the limitations associated with its clinical application. Finally, we outline future perspectives in OA research, emphasizing its dual role as both an antioxidant and an anticancer agent, and we suggest potential directions for further preclinical and clinical investigations aimed at translating these findings into effective therapeutic interventions.

## 2. Oxidative Stress in Carcinogenesis

A free radical is defined as any molecular species possessing one or more unpaired electrons in its outer orbitals, which renders it highly reactive and unstable. Such species can act either as oxidants by donating an electron, or as reductants by accepting one, thereby engaging in various redox reactions within biological systems. Free radicals are continuously generated as byproducts of normal physiological processes, including aerobic metabolism, mitochondrial electron transport, and immune defense mechanisms such as phagocytosis [16]. In addition, exogenous factors, including ionizing radiation, air pollutants, xenobiotics, and cigarette smoke, represent important sources of radical generation [17].

**Figure 2:**
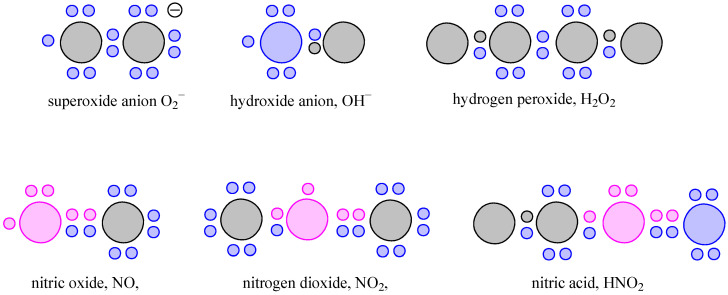
The most common Reactive Oxygen Species and Nitrogen Reactive Species.

**Figure 3:**
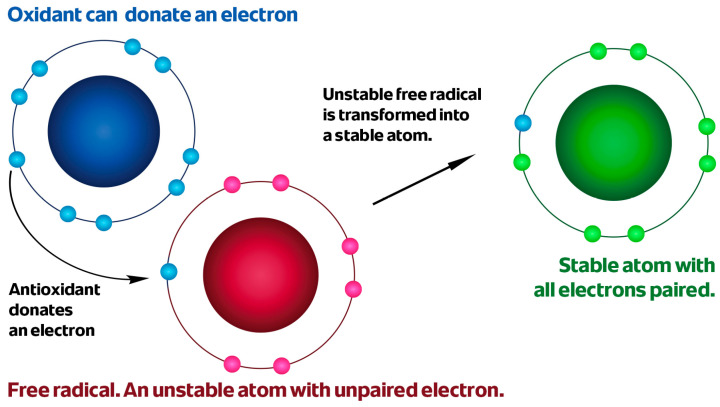
The mechanism of antioxidant action via electron donation.

Reactive Oxygen Species constitute a major subclass of free radicals that specifically involve oxygen-containing molecules. Reactive Nitrogen Species are a group of molecules, derived from nitric oxide (NO). The most common ROS and RNS are presented in Figure 2. Free radicals represent a broad class of molecular species characterized by the presence of one or more unpaired electrons and are a type of ROS or RNS. The presence of unpaired electron (or electrons) confers high chemical reactivity and instability of free radicals [16]. Antioxidants counteract the harmful effects of free radicals by neutralizing their unpaired electrons, thereby preventing uncontrolled chain reactions that lead to oxidative damage. The mechanism of antioxidant action via electron donation is schematically illustrated in Figure 3.

**Figure 4:**
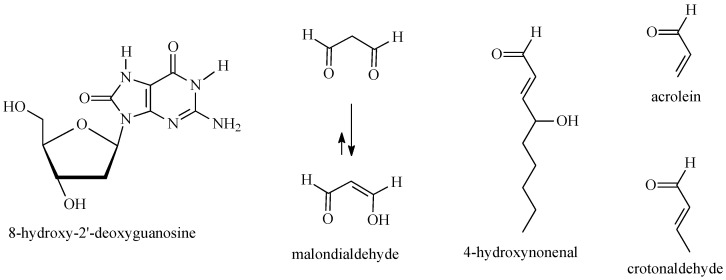
Biomarkers of oxidative stress.

ROS and RNS play a dual role in cell biology—at low concentrations, they are important mediators of signaling, proliferation, and differentiation [4], whereas in excess they lead to lipid peroxidation, protein oxidation, and DNA damage, promoting genomic instability and neoplastic transformation [18,19]. Chronic oxidative stress manifests biochemically through the accumulation of “molecular fingerprints” of damage, which can be detected using specific biomarkers such as 8-hydroxy-2′-deoxyguanosine (IUPAC name: 2-amino-9-[(2*R*,4*S*,5*R*)-4-hydroxy-5-(hydroxymethyl)oxolan-2-yl]-1,7-dihydropurine-6,8-dione), 4-hydroxynonenal (IUPAC name: (*E*)-4-hydroxynon-2-enal), acrolein (IUPAC name: prop-2-enal), crotonaldehyde (IUPAC name: (*E*)-but-2-enal) or malondialdehyde (IUPAC name: propanedial) (Figure 4) [20]. These biomarkers are widely used in carcinogenesis research to evaluate the degree of ROS/RNS-induced damage [20].

Cancer cells are characterized by elevated ROS levels due to their accelerated metabolism and altered redox homeostasis, which paradoxically can be exploited therapeutically [21]. Elevated ROS levels in cancer cells increase their susceptibility to further oxidative damage and form the basis of therapeutic strategies that selectively increase ROS to induce apoptosis [22]. Exceeding the tolerance threshold to oxidative stress leads to cancer cell death, whereas healthy cells are better able to cope with the pro-oxidant burden. This fact forms the basis of the concept of the “tolerance threshold in cancer therapy” [23].

### 2.1. Oxidative Eustress in Cancerogenesis

Physiological levels of reactive oxygen and nitrogen species, referred to as oxidative eustress, comprise low to moderate concentrations of oxidants that regulate biochemical processes such as carboxylation, hydroxylation, and modulation of signaling pathways [24]. In line with current recommendations, we here refer to low-to-moderate oxidant levels as physiological redox signaling (formerly termed ‘oxidative eustress’), which must be clearly distinguished from oxidative distress associated with disrupted redox control and molecular damage. When ROS/RNS levels exceed this physiological range (oxidative distress), they activate redox-sensitive transcription factors and pro-inflammatory genes, thereby intensifying inflammation and disease progression [25]. In cancer cells, elevated ROS levels promote genomic instability, the selection of mutant clones, and treatment resistance [26], while chronic inflammation additionally drives carcinogenesis [24]. Modulation of the redox state and control of inflammation, including with the application of oleanolic acid, represent a promising therapeutic strategy in oncology [24].

In cancer therapy, tumor-associated macrophages also exhibit pleiotropic biological properties, capable of both promoting and inhibiting tumor development depending on the modulatory effects of tumor cells on the immune system [27]. It is important to highlight that disturbances in the redox balance of tumor cells may promote uncontrolled proliferation and enhanced invasive potential, both of which are key contributors to cancer progression [28]. Moreover, disruption of redox homeostasis can activate pro-tumorigenic signaling cascades, including the Nrf2 pathway (nuclear factor erythroid 2-related factor 2), which—despite its well-recognized cytoprotective role—may paradoxically support cancer cell survival and therapy resistance by upregulating antioxidant and detoxifying enzymes [29]. Transient Nrf2 activation in normal tissues exerts tumor-suppressive effects by inducing antioxidant and detoxifying enzymes. By contrast, persistent Nrf2 activation in cancer cells, often driven by alterations in the KEAP1/NRF2/CUL3 axis, enhances stress resistance, promotes metabolic rewiring and contributes to chemoresistance, so that several malignancies are now considered “Nrf2-addicted”. This dualistic function of Nrf2 in carcinogenesis underscores the necessity of a nuanced understanding of its regulation, taking into account cellular context and tumor-specific characteristics. Consequently, there is a growing need for precise therapeutic approaches capable of selectively modulating this pathway in malignant cells while minimizing unintended effects on normal tissues. As a result, Nrf2-oriented interventions have become an active area of research, with efforts focused on inhibiting its pro-tumor activity while leveraging its protective roles in healthy cells [30].

Nrf2 activation and NF-κB (nuclear factor kappa B cells) inhibition can protect healthy cells from carcinogenesis, but in cancer cells they promote resistance to chemo- and radiotherapy [30]. Therefore, oncological therapies aim to modulate these pathways to weaken tumor defense mechanisms while protecting healthy tissues. NF-κB and AKT (AKT—protein kinase B, known as PKB) with their pro-survival functions, require selective blocking to increase treatment efficacy [31]. Multi-target substances, such as oleanolic acid, can act as pro-oxidants in cancer cells and antioxidants in healthy cells, which helps reduce the side effects of therapy [32]. Thus, oleanolic acid may activate Nrf2 and antioxidant defenses predominantly in healthy tissues, while in tumor cells it exerts mainly pro-oxidant and pro-apoptotic effects, particularly when combined with inhibition of NF-κB and related survival pathways. Modulation of NF-κB, a key regulator of pro- and anti-apoptotic and inflammatory processes, is an important target of anticancer therapy. The potential applications of NF-κB pathway inhibition in anticancer chemotherapy are in the early stages of research, but such approaches promise to increase the efficacy of cancer treatment [30].

### 2.2. DNA Damage and Deregulation of Signaling Pathways by ROS/NOS

Excessive ROS and RNS cause DNA damage, mutations, and genomic instability, which are central to cancer initiation and progression [28]. Oxidative stress also disrupts metabolic and signaling pathways and promotes chronic inflammation [33]. Mitochondria are highly vulnerable to oxidative injury, and their dysfunction further increases ROS production [34].

Elevated ROS can mutate oncogenes and tumor suppressor genes [35], activate MAPK (mitogen-activated protein kinase) and PI3K/AKT (phosphatidylinositol 3-kinase/AKT) signaling [4], and destabilize the genome and epigenome [36,37]. Excessive ROS production also activate pro-tumorigenic transcription factors, including NF-κB, AP-1 (activator protein 1), and STAT3 (signal transducer and activator of transcription 3), enhancing proliferation and inflammation. Chronic inflammation and gut microbiota dysbiosis further amplify ROS levels, reinforcing oncogenic processes [38].

Dysregulation of cellular stress signaling can activate mitogen-activated protein kinase pathways, influencing cell survival and death. Excessive stress combined with an inadequate adaptive response may promote neoplastic transformation, indicating an inverse relationship between the activation threshold of stress responses and cancer risk [39]. While moderate stress (eustress) can enhance resistance to oxidative damage, prolonged distress leads to the accumulation of harmful metabolites, redox imbalance, and progressive molecular and tissue damage, ultimately facilitating uncontrolled cancer cell proliferation and invasion [40]. In addition, persistent endoplasmic reticulum (ER) stress—often driven by the accumulation of misfolded proteins—disrupts calcium homeostasis and increases ROS levels, thereby activating autophagy and apoptosis pathways and contributing further to carcinogenesis [41].

It is well established that many cancers exhibit constitutive activation of PI3K/AKT family kinases, which increases cell survival, proliferation, and resistance to therapy. Endoplasmic reticulum (ER) stress can further influence tumor progression by modulating AKT activity in a manner dependent on the severity and duration of the stress response [42]. Chronic ER stress with sustained activation of the unfolded protein response, triggers NF-κB- and JNK-dependent production of pro-inflammatory cytokines and reactive oxygen species, thereby creating a persistent inflammatory–oxidative environment conducive to cancer development and progression [43].

Chronic activation of the tumor suppressor protein p53, a central regulator of the cell cycle and apoptosis, is critical for orchestrating cellular responses to stress and remains a prominent target in anticancer strategies. Both environmental and endogenous sources of reactive oxygen species (ROS), such as ultraviolet radiation, chemical pollutants, and metabolic by-products, can elevate intracellular ROS levels. Excessive ROS promotes the accumulation of DNA lesions, which in turn activates p53-dependent stress responses aimed at maintaining genomic integrity. However, when p53 function is compromised, the capacity of cells to detect and repair ROS-induced DNA damage is diminished. This impairment exacerbates the accumulation of genomic alterations and fosters conditions conducive to neoplastic transformation, thereby amplifying the contribution of ROS and p53 dysfunction to tumor development [44,45].

Endoplasmic reticulum stress, associated with PI3K/AKT activation, can inactivate p53 through MDM2-mediated (Mouse Double Minute 2 homolog-mediated) degradation, which together with gene TP53 (tumor protein p53 gene) mutation promotes treatment resistance [46]. Restoring p53 function (e.g., by zinc) is a promising strategy in cancer treatment. The complex interconnections of oxidative stress, inflammation, epigenetics, and cell signaling require multitargeted approaches, including the use of natural compounds such as oleanolic acid [47].

## 3. Physicochemical Profile and Natural Sources of Oleanolic Acid

### 3.1. Structure and Stereochemistry of Oleanolic Acid

**Figure 5:**
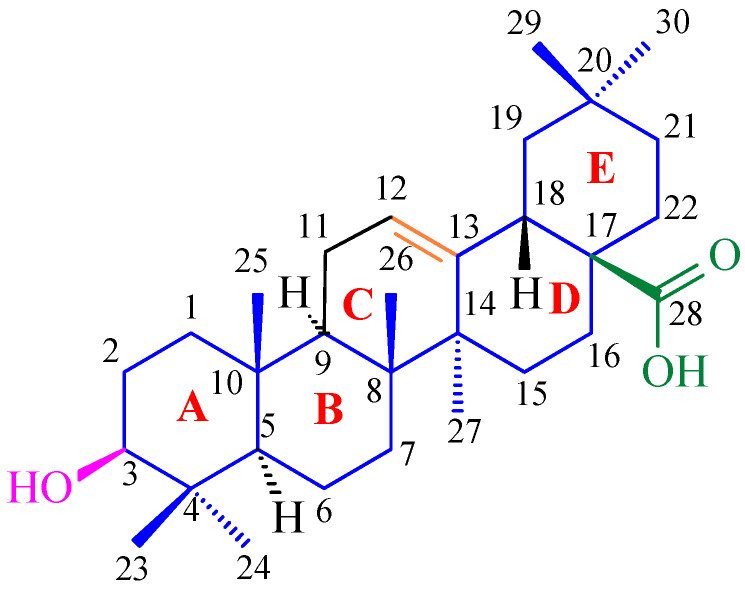
Structure of oleanolic acid (OA). Chiral carbon atoms of OA molecule: C-3, C-5, C-8, C-9, C-10, C-14, C-17 and C-18.

Oleanolic acid is a triterpenoid compound found in numerous plant species and characterized by a specific chemical structure. Its molecular formula is C_30_H_48_O_3_ and its molecular weight is 456.7 g/mol (grams per mole) indicating a relatively complex molecule. It is a triterpene composed of five six-carbon rings (Figure 5), giving it a characteristically rigid structure [48]. This unique molecular architecture determines its ability to form diverse interactions with biomolecules, which is crucial for its biological activity [48]. The oleanolic acid ring contains a hydroxyl group at the C-3 (Figure 5), a carboxyl group at the C-17, and a double bond between the C-12 and the C-13, enabling numerous chemical modifications and the synthesis of derivatives with enhanced activity, that we proved earlier (e.g., [49]). These modifications may include esterification, etherification, oxidation, reduction, or adduct formation, allowing for fine-tuning of the molecule’s physicochemical and pharmacological properties.

**Figure 6:**
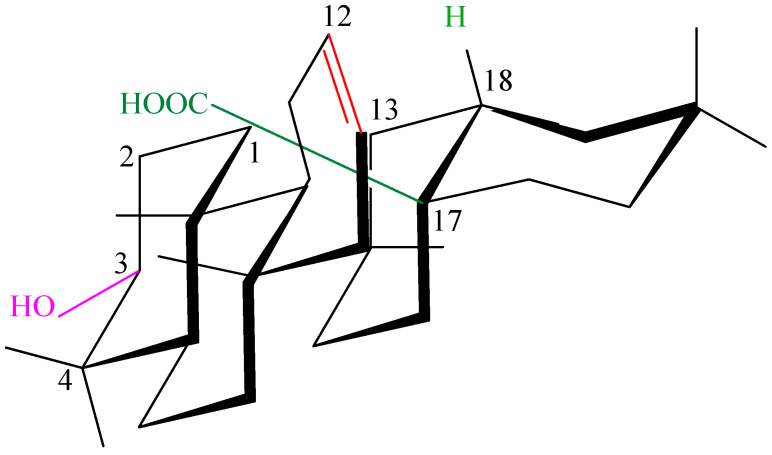
Conformational structure of oleanolic acid.

The stereochemistry of oleanolic acid is strictly defined (Figure 6), which influences its interactions with biological receptors and enzymes, and any changes in its spatial configuration can significantly modify its activity [13].

Furthermore, the lipophilic nature of oleanolic acid, resulting from the predominance of hydrocarbon segments in its structure, determines its low water solubility, which poses a challenge in pharmaceutical formulations but also facilitates interactions with cell lipid membranes. These properties, although beneficial for interactions with molecular targets in a lipophilic environment, create barriers to achieving adequate therapeutic concentrations of oleanolic acid or its derivatives in target tissues after oral administration, requiring strategies to optimize the bioavailability of this triterpene. Furthermore, its hydrophobic nature results in low chromatographic mobility, which is reflected in low retention coefficients in chromatographic systems with polar media, such as silica gel [49,50]. However, despite these pharmacokinetic challenges, the high selectivity of oleanolic acid in interacting with lipid components of cell membranes and its ability to stabilize membrane structure make it a promising candidate for further investigation in its mechanisms of anticancer action [51].

An isomer of oleanolic acid, ursolic acid, has a similar structure but differs in the spatial arrangement of the methyl group at the C-20, which contributes to subtle differences in their biological activity and interactions with molecular targets. These structural nuances between oleanolic and ursolic acids are crucial for understanding their specific actions in the body, allowing for precise targeting of their therapeutic applications [13]. These differences manifest at the level of affinity for enzymes, receptors, and transporter proteins, which consequently influences different bioavailability and toxicity profiles, providing a basis for the rational design of new compounds with targeted pharmacological action [13].

### 3.2. Endogenous Occurrence and Major Plant Sources

The biosynthesis of oleanolic acid in plants begins with the cyclization of 2,3-oxidosqualene, a precursor produced in the mevalonate pathway, which highlights the affiliation of this triterpene to a broad family of terpenoid natural products, including sterols and triterpenoid saponins [51]. This ubiquity in the plant kingdom reflects its role in secondary metabolism and plant defense mechanisms, and also confirms its importance as a component of the human diet [52].

Oleanolic acid is widely available in many medicinal and edible plants, occurring both as free acid and as aglycones for triterpenoid saponins [53]. Numerous review publications presenting the popularity of oleanolic acid in the plant kingdom are known in the scientific literature with the most significant by Yeung [14]. Numerous publications are also known in which the authors demonstrate the presence of oleanolic acid in individual plants and present the results of studies on various areas of pharmacological activity of plant extracts containing oleanolic acid or pure oleanolic acid isolated from plants. Several examples of publications regarding various sources of oleanolic acid are presented below.

The chemical composition of ethanolic olive leaf extracts has attracted considerable scientific interest, largely due to the substantial presence of bioactive constituents such as phenolic derivatives, alditols, and pentacyclic triterpenes—each recognized for noteworthy pharmacological activities [54]. Among these compounds, oleanolic acid is the predominant triterpene, typically accounting for approximately 3.0–3.5% of the dry weight. It is accompanied by notable amounts of maslinic acid, as well as smaller quantities of ursolic acid, erythrodiol, and uvaol [55].

Apple (*Malus domestica*, Mill.), pear (*Pyrus communis* L.), and quince (*Cydonia oblonga* Mill.) leaves have also been shown to be valuable sources of phenolic compounds and triterpenes, expanding the spectrum of potential sources of oleanolic acid. The content of oleanolic acid in these sources was from 10 to 20 mg/100 g dried apple fruits, from 18 to 30 mg/100 g dried pear fruits and from 2 to 19 mg/100 mg dried quince fruits [56]. Furthermore, oleanolic acid is present in significant amounts in mistletoe, a plant traditionally used in folk medicine, confirming its medicinal value. The concentration of OA in dried leaves of mistletoe was 8.52 mg/g dry material [57]. Yunoki and co-workers investigated the content of oleanolic acid (OA) in pomace, the solid residue generated during wine production from various cultivars of Kiyomi oranges (e.g., Citrus reticulata Blanco × Citrus × aurantium L.). Their analysis demonstrated that this byproduct represents a valuable natural source of OA. This triterpene was present in pomace from Kiyomi oranges in the amount of 110 mg/g dried fruit pomace [58]. Vegetable oils, particularly olive oil, contain oleanolic acid in the unsaponifiable fraction, which contributes to their health-promoting properties and is an important element of the Mediterranean diet, known for its beneficial effects on health. Common olive oil contains oleanolic acid in the amount 5.2 mg/g dry extract from olive fruits [59].

Additionally, the presence of oleanolic acid has also been found in walnut (*Juglans regia* L.) kernels, where it contributes to its antioxidant properties, increasing the availability of this valuable compound in various dietary sources. The content of oleanolic acid in its saponin form was from 95 to 141 mg/g dry walnut extract [60].

## 4. Key Antioxidant Mechanisms of Oleanolic Acid

Oleanolic acid exhibits complex and multifaceted antioxidant activity, encompassing both direct free radical scavenging and modulation of endogenous defense systems, which is crucial for its therapeutic potential in diseases associated with oxidative stress. Its antioxidant activity stems from its unique chemical structure, which allows it to neutralize reactive oxygen species and reactive nitrogen species, thereby minimizing damage to biomolecules. This ability makes it a promising candidate for preventive and therapeutic strategies for many pathologies, including cancer [61].

The antioxidant mechanisms of oleanolic acid (as well as other chemical compounds) can be classified into two major categories: (i) direct antioxidant activities and (ii) indirect transcriptional or enzyme-mediated mechanisms [62]. The first category includes mechanisms such as: direct scavenging of free radicals, activation of endogenous antioxidant systems, stabilization of cell membranes and inhibition of lipid peroxidation, and chelation of transition metal ions. The second group of antioxidant mechanism comprises processes such as: inhibition of pro-oxidant and pro-inflammatory enzymes, downregulation of NF-κB and MAPK, mitochondrial protection, and HO-1 induction. The mentioned types of mechanisms are briefly characterized below.

In our review article [13], we have systematically collected and critically discussed studies addressing the specific mechanisms underlying the antioxidant activity of oleanolic acid. By integrating current findings, this work highlights the molecular pathways through which oleanolic acid exerts its effects, thereby providing a comprehensive basis for further experimental and clinical investigations into its therapeutic potential.

### 4.1. Direct Scavenging of Free Radicals (OH^•^, O_2_^•−^, ^1^O_2_)

This mechanism involves the direct neutralization of reactive oxygen species (ROS), such as hydroxyl radicals (^•^OH), superoxide anions (O_2_^•−^), or singlet oxygen (^1^O_2_), by donating hydrogen atoms or electrons, thus stabilizing unstable molecules and preventing oxidative damage. Due to its chemical structure, oleanolic acid effectively reacts with radicals, transforming them into less reactive forms, which minimizes their ability to damage biological macromolecules [63,64]. In carcinogenesis, the ability of oleanolic acid to limit ROS-induced DNA and protein damage is particularly important because it minimizes the risk of mutations and protein dysfunctions that can lead to neoplastic transformation [64]. Representative results of studies evaluating the antioxidant activity of oleanolic acid are presented in Table 1.

### 4.2. Activation of Endogenous Antioxidant Systems

This complex mechanism involves the induction of the transcription factor Nrf2, which regulates the expression of genes encoding antioxidant enzymes such as superoxide dismutase, catalase, and glutathione peroxidase. Activation of the Nrf2 pathway by oleanolic acid leads to increased production of these enzymes, which enhances endogenous cellular defense against oxidative stress. Additionally, oleanolic acid can also influence the activity of other transcription factors involved in the response to oxidative stress, demonstrating its ability to modulate a wide range of signaling pathways [64].

These enzymes, such as superoxide dismutase, catalase, and glutathione peroxidase, play a key role in the detoxification of reactive oxygen species, converting them into less harmful products and maintaining cellular redox homeostasis. Enhancing the expression of these enzymes by OA contributes to more effective neutralization of excess free radicals, thus reducing oxidative damage in tissues. Importantly, available data indicate that Nrf2 activation by oleanolic acid is transient and mainly cytoprotective in non-transformed cells, whereas in cancer cells OA is usually used at cytotoxic, pro-oxidant concentrations and simultaneously inhibits pro-survival pathways such as NF-κB, PI3K/AKT/mTOR or STAT3. In this context, the pro-apoptotic and pro-oxidant effects of OA in tumor cells are likely to outweigh any potential pro-survival influence of Nrf2 [65].

### 4.3. Stabilization of Cell Membranes and Inhibition of Lipid Peroxidation

Due to its chemical structure, oleanolic acid can integrate into the lipid bilayer of cell membranes, contributing to their stabilization and reducing their susceptibility to free radical attacks. This mechanism is particularly important in the context of preventing oxidative damage, as lipid peroxidation is a key step in the initiation of many pathologies associated with oxidative stress [64]. The ability of oleanolic acid to inhibit lipid peroxidation also results from direct free radical scavenging, effectively preventing the formation of toxic aldehydes, such as malondialdehyde, which are markers of oxidative damage [66].

Additionally, oleanolic acid’s stabilization of cell membranes may influence the integrity of cellular organelles such as mitochondria, protecting them from oxidative damage and dysfunction, which is crucial for maintaining cellular homeostasis. Its ability to interact with membrane phospholipids limits the propagation of free radical reactions, which is crucial for protecting cells from damage induced by reactive oxygen species [67]. Oleanolic acid has been shown to potently inhibit lipid peroxidation in rat liver microsomes, acting as an effective inhibitor of this process, which is comparable to or even better than some commercial antioxidants [64].

### 4.4. Chelation of Transition Metal Ions

This property of oleanolic acid involves binding metal ions, such as iron and copper, which catalyze the formation of highly reactive hydroxyl radicals in the Fenton reaction, effectively reducing the production of these harmful oxidants [64,67]. Representative results of studies evaluating the antioxidant activity of oleanolic acid are presented in Table 1 [64,68]. The ability to chelate transition metals is particularly important in the context of preventing DNA and protein damage, which can lead to mutations and neoplastic transformation. This mechanism is crucial for maintaining redox homeostasis in the cell, which is the foundation of protection against oxidative stress induced by heavy metals [69].

**Table 1:** Representative results of studies evaluating the antioxidant activity of oleanolic acid.

OA Concentration	Activity	Results	Ref.
1 mg/mL (0.1%) in DMSO	Free radical-scavenging activity (DPPH)	2.9%	[64]
	Hydroxy radical-scavenging activity	33.4%	[64]
	Superoxide anion radical-scavenging activity	26.3%	[64]
	Inhibition of lipid peroxidation in rat liver microsomes induced by vitamin C/Fe^2+^	10.7%	[64]
	Inhibition of lipid peroxidation in rat liver microsomes induced by CHP	17.0%	[64]
	Inhibition of lipid peroxidation in rat liver microsomes induced by CCl_4_/NADPH	17.0%	[64]
10 μg/mL (0.001%) in methanol	Superoxide anion radical-scavenging activity	30.25%	[68]
	Hydroxy radical-scavenging activity	25.45%	[68]
	Nitric oxide radical scavenging	11.15%	[68]

### 4.5. Inhibition of Pro-Oxidant and Pro-Inflammatory Enzymes

By inhibiting the activity of pro-oxidant enzymes such as NADPH oxidase (nicotinamide adenine dinucleotide phosphate oxidase) and pro-inflammatory enzymes such as matrix metalloproteinases (MMPs), mainly MMP-2 and MMP-9, oleanolic acid directly limits the generation of reactive oxygen and nitrogen species and reduces oxidative stress-induced inflammatory processes that contribute to disease progression. Reducing the activity of these enzymes is crucial for preventing tissue damage and modulating the tumor microenvironment, which in turn limits the growth and spread of cancer cells [70].

Inhibition of NADPH oxidase leads to a reduction in superoxide anion levels, which is important for maintaining redox balance and preventing lipid peroxidation, DNA damage, and protein modifications in cells. Furthermore, regulation of MMPs by OA may reduce the invasiveness of cancer cells and their ability to metastasize by inhibiting extracellular matrix degradation. Blocking these inflammatory and prooxidant enzymes contributes to a global reduction in inflammation, which is crucial in the context of chronic inflammation, which is a recognized risk factor for carcinogenesis [71].

### 4.6. Downregulation of NF-κB and MAPK

This mechanism involves the modulation of key signaling pathways, such as the transcription factor NF-κB and mitogen-activated protein kinases, both of which play central roles in regulating cellular stress and inflammatory responses. Oleanolic acid (OA) has been shown to inhibit these pathways, resulting in the downregulation of pro-inflammatory and pro-oxidative gene expression and thereby strengthening its antioxidant activity. Through this regulatory effect, OA contributes to a reduction in pro-inflammatory cytokine levels, which is of critical importance in the prevention and treatment of cancer [64].

Activation of NF-κB is frequently associated with the upregulation of genes involved in inflammation, apoptosis, cell proliferation, angiogenesis, and metastasis [72]. Thus, inhibition of this pathway by oleanolic acid is particularly relevant for limiting tumor progression and enhancing the sensitivity of cancer cells to chemotherapy [64].

Beyond NF-κB, OA also targets MAPK pathways, which are essential regulators of cell growth, differentiation, and apoptosis, further reinforcing its therapeutic potential in chemoprevention and cancer treatment. Moreover, pentacyclic triterpenes, including OA, have been reported to suppress tumor angiogenesis and promote differentiation of cancer cells, thereby contributing to the restriction of tumor growth and metastasis.

Altogether, the ability of oleanolic acid to modulate multiple signaling pathways and to interfere with inflammatory and angiogenic processes underscores its multitargeted mode of action, positioning it as a promising candidate for further exploration in oncology [64].

### 4.7. Other Mechanisms—Mitochondrial Protection, HO-1 Induction

Other mechanisms of antioxidant activity of oleanolic acid include its ability to protect mitochondrial integrity, which is crucial for maintaining normal cellular function and preventing the generation of mitochondrial reactive oxygen species [73]. Furthermore, OA can induce the expression of heme oxygenase-1 (HO-1), a protein with potent antioxidant and anti-inflammatory properties, contributing to cell protection from oxidative stress [74].

This synergy of antioxidant, anti-inflammatory, and pro-apoptotic effects highlights the multifaceted potential of oleanolic acid in the fight against cancer.

## 5. Interconnection with Carcinogenesis Pathways

### 5.1. ROS-Mediated DNA Damage and Mutagenesis as Drivers of Tumor Initiation

DNA damage induced by ROS represents one of the best-characterized molecular mechanisms underlying cancer initiation. Under physiological conditions, cells possess efficient antioxidant and repair systems that maintain redox balance and genomic integrity. However, prolonged exposure to conditions that promote oxidative stress—such as chronic inflammation, metabolic disorders, xenobiotic exposure or ionizing radiation—leads to excessive ROS production and accumulation of DNA damage [75,76]. ROS interact with both purine and pyrimidine bases, generating numerous oxidation products [77,78]. Free radicals also induce DNA strand breaks (both single- and double-stranded), promote the formation of interstrand crosslinks, and cause chromatin conformational changes. If such damage is not repaired by DNA repair systems such as BER (base excision repair), NER (nucleotide excision repair), HR (homologous recombination), or NHEJ (non-homologous end joining), mutations become fixed and disrupt the expression of genes regulating the cell cycle, apoptosis, and DNA repair. As a consequence, proto-oncogenes may become activated or tumor suppressor genes inactivated, triggering the process of neoplastic transformation [79,80,81].

Furthermore, ROS can influence epigenetic processes—oxidative modifications of nitrogenous bases may lead to abnormal DNA methylation, while histone protein damage alters chromatin structure and gene accessibility to transcription factors. Such epigenetic changes, even in the absence of direct mutations, can disturb the regulation of cellular proliferation, differentiation, and apoptosis [82,83].

In addition to epigenetics, ROS contribute to mutagenesis and facilitate the survival and clonal expansion of cells characterized by genomic instability, promoting the development of cancer [84].

In summary, oxidative stress driven by ROS is one of the main factors initiating carcinogenesis, through the induction of DNA damage, mutagenesis, epigenetic alterations, and activation of signaling pathways that favor cellular transformation.

### 5.2. Suppression of Oxidative Inflammation and Modulation of Transcription Factors Relevant to Cancer Progression

Oleanolic acid modulates oxidative and inflammatory processes by interacting with signaling pathways and transcription factors involved in cancer progression. Studies show that OA not only inhibits the activation of pro-inflammatory mediators, but also alters the balance between pro- and anti-proliferative signaling in cancer cells. These activities involve regulation of transcription factors, positioning OA as a modulator of the tumor microenvironment and a promising complementary agent to conventional anticancer therapy.

Hwang et al. demonstrated that OA suppresses NF-κB signaling through downregulation of MAFK (v-maf avian musculoaponeurotic fibrosarcoma oncogene homolog K) expression, revealing a novel anti-inflammatory mechanism [85]. In LPS-stimulated (lipopolysaccharides-stimulated) RAW 264.7 macrophages (macrophage-like, Abelson leukemia virus-transformed cell line), OA significantly decreased nitric oxide and prostaglandin E_2_ (PGE_2_), showing greater efficacy than other major compounds from *Prunella vulgaris* var. lilacina. Downregulation of MAFK reduced p65 (subunit of the NF-κB transcription factor) acetylation and altered IκBα (inhibitor of kappa B alpha) phosphorylation, ultimately inhibiting NF-κB activation. Concurrently, OA induced Nrf2-dependent cytoprotective genes, such as HO-1 and NQO1 (NAD(P)H quinone oxidoreductase 1), highlighting its dual ability to attenuate oxidative inflammation and enhance antioxidant defenses [85].

Subsequent in vivo studies confirmed that NF-κB inhibition is also relevant in lung inflammation. In a Whistar rat model of silicosis, OA administration significantly decreased pro-inflammatory cytokines, including TNF-α (tumor necrosis factor α) and TGF-β1 (tumor growth factor β1), and reduced collagen I and III deposition in lung tissue. OA also decreased phosphorylated AKT1 (AKT serine/threonine kinase 1) levels, leading to NF-κB inhibition and attenuation of silica-induced pulmonary inflammation. In addition, OA restored redox homeostasis by modulating malondialdehyde concentration and by increasing the activity of antioxidant enzymes, superoxide dismutase and glutathione peroxidase [86].

Complementary in vitro studies have confirmed that OA-mediated suppression of oncogenic signaling extends beyond macrophage and lung models. In human bladder cancer (T-24) cells, OA treatment inhibited cell proliferation, with the strongest effect observed at 50 μM. OA also increased apoptotic cell numbers and caspase-3 activity. On the molecular level, OA reduced phosphorylated AKT, leading to inhibition of mTOR (mammalian target of rapamycin), a central regulator of cancer cell growth, survival, and chemoresistance. OA also decreased ERK1/2 (Extracellular signal-Regulated Kinases 1 and 2) phosphorylation, further reducing cancer cell proliferation [87].

OA additionally exhibits anti-angiogenic properties. Administration of OA in a mouse model of colorectal cancer significantly inhibited tumor growth and reduced tumor vascularization. The colorectal tumors were induced in male BALB/c (albino, laboratory-bred strain of the house mouse) nude mice by subcutaneous implantation of HT-29 cancer cells (human colorectal carcinoma cells), after which the animals were treated with OA once daily, six days per week, for sixteen days. Tumor volume in OA-treated mice was markedly smaller than in the control group, and immunohistochemical analysis revealed a significant decrease in microvessel density identified by the endothelial marker CD31 (platelet endothelial cell adhesion molecule, known as PECAM-1). In vitro, OA inhibited proliferation, migration, and capillary tube formation of human umbilical vein endothelial cells in a dose- and time-dependent manner. At the molecular level, OA markedly suppressed the activation of the STAT3 and sonic hedgehog (SHH) signaling pathways—it reduced IL-6 (interleukin-6) STAT3 phosphorylation and decreased the expression of SHH and GLI-1 (glioma-associated oncogene homolog 1) both in HT-29 colorectal cancer cells and in tumor tissues. Simultaneously, OA downregulated the expression of the pro-angiogenic genes VEGF-A (vascular endothelial growth factor A) and bFGF (basic fibroblast growth factor), which are key effectors of these pathways. These findings indicate that oleanolic acid inhibits colorectal cancer growth and angiogenesis by concurrently suppressing the STAT3 and SHH signaling pathways and limiting the expression of their downstream target genes [88].

Collectively, studies [85,86,87,88] demonstrate a consistent mechanistic profile in which OA regulates inflammatory and proliferative signaling networks across diverse biological models. OA inhibits NF-κB activation while inducing Nrf2-dependent antioxidant defenses, attenuates AKT/mTOR and ERK1/2 oncogenic signaling, promotes apoptosis, and suppresses tumor angiogenesis by downregulating STAT3 and SHH pathways. These mechanisms establish OA as a strong candidate for therapeutic strategies targeting inflammation-associated and cancer-related diseases.

### 5.3. Crosstalk Between Antioxidant Signaling and Oncogenic Pathways (e.g., NF-κB vs. Nrf2 Balance)

Crosstalk is a process involving the interaction between different signaling pathways within a cell. This means that proteins shared by two or more pathways can cause the activity of one pathway to influence the activity of another. As a result, the cell does not respond to stimuli in an isolated manner but instead integrates various environmental signals, allowing for a more complex and precise biological response [89,90]. In the context of cancer biology, oxidative stress exemplifies a potent modulator of signaling crosstalk. ROS, acting as second messengers, influence multiple signaling cascades simultaneously—including mitochondrial apoptosis pathways, endoplasmic reticulum stress responses, and extrinsic death receptor signaling. This convergence enables ROS to coordinate diverse cellular outcomes such as proliferation, differentiation, or programmed cell death [91].

One particularly well-studied example of signaling crosstalk involves the transcription factors NF-κB and Nrf2, which regulate inflammation and oxidative stress responses, respectively. These pathways do not operate in isolation; instead, they dynamically influence one another through multiple molecular mechanisms. For instance, NF-κB can suppress Nrf2 activity by competing for the transcriptional coactivator CBP/p300 (CREB-binding protein, CBP and p300, or EP300, transcriptional coactivators) or by recruiting histone deacetylase 3 (HDAC3), which inhibits Nrf2-dependent gene transcription. Conversely, Nrf2 activation—especially via its downstream target HO-1—can dampen NF-κB signaling and reduce the expression of proinflammatory cytokines such as TNF-α and IL-1β (interleukin-1 beta). This reverse regulation exemplifies how crosstalk enables cells to fine-tune their responses to complex stimuli like oxidative damage or drug-induced stress. Additionally, common regulatory proteins like GSK3β (glycogen synthase kinase 3 beta) and β-TrCP (beta-transducin repeat-containing protein) control both pathways, making their interaction even more complex. GSK3β phosphorylates Nrf2, targeting it for degradation, while also influencing NF-κB activity depending on cellular context [92,93].

Understanding the Nrf-2–NF-κB crosstalk opens avenues for dual-targeted therapeutic strategies, especially using phytochemicals like OA. This compound has been shown to simultaneously activate Nrf2 and inhibit NF-κB signaling, thereby enhancing antioxidant defenses while reducing inflammation. Beyond these two pathways, OA also modulates other signaling cascades involved in cell survival, apoptosis, and metabolic regulation pathways. This multifaceted activity underscores OA’s potential as a pleiotropic regulator capable of orchestrating complex cellular responses through extensive crosstalk among multiple signaling networks.

## 6. Anticancer Mechanisms Mediated by Oxidative Stress Modulation

### 6.1. OA-Induced Apoptosis (via Caspases, BAX/BCL-2 Signaling)

Oleanolic acid exerts proapoptotic activity through activation of both intrinsic and extrinsic apoptosis pathways. It promotes cytochrome c release from mitochondria, activates caspases 3, 8, and 9, and increases the BAX/BCL-2 ratio, reflecting the balance between pro- and anti-apoptotic proteins [87,94,95]. OA also engages the ROS/ASK-1/p38 MAPK pathway (ASK-1, apoptosis signal-regulating kinase 1), resulting in phosphorylation of pro-apoptotic proteins such as BAX and BIM (BCL-2–interacting mediator of cell death) and concomitant inactivation of BCL-2.

In cancer cells, OA enhances sensitivity to TRAIL (TNF-related apoptosis-inducing ligand) by upregulating death receptors DR4 and DR5 and promoting DISC (death-inducing signaling complex) formation, which leads to caspase-8 activation and initiation of apoptosis. OA further inhibits survival pathways, including PI3K/AKT/mTOR and NF-κB, thereby shifting cancer cells toward programmed cell death. Additionally, OA can induce ROS-dependent autophagy, which may act synergistically with apoptosis to eliminate malignant cells [94,96].

Recent studies have expanded understanding of OA’s translational potential. Zhang et al. reported that OA inhibits proliferation of the human leukemia cell line HL-60 in a dose- and time-dependent manner via apoptosis. Morphological analysis, DNA fragmentation, and flow cytometry confirmed classic apoptotic features, including chromatin condensation, apoptotic body formation, and an increase in the sub-G1 population. Western blot analysis demonstrated activation of caspase-9 and caspase-3, as well as PARP (poly(ADP-ribose) polymerase) cleavage, indicating involvement of both mitochondrial and receptor-mediated pathways [97].

Importantly, this mechanism was not limited to hematological malignancies. Similarly, Hu et al. reported that OA significantly inhibits proliferation and viability of human colorectal carcinoma HCT-116 and SW-480 cells, inducing both apoptosis and autophagy. This effect is associated with AMPK (AMP-activated protein kinase) pathway activation and mTOR inhibition, leading to increased expression of pro-apoptotic genes (BAX, caspases-3, -8, -9) and autophagy-related proteins (Beclin 1, LC3B-II, and ULK1; Beclin 1—protein that in humans is encoded by the BECN1 gene, LC3B-II—lipidated form of the protein LC3B—microtubule-associated protein 1 light chain 3 Beta, and ULK1—serine/threonine kinase that plays a central, initiating role in autophagy), while anti-apoptotic genes (mTOR, BCL-2) are downregulated [98].

**Figure 7:**
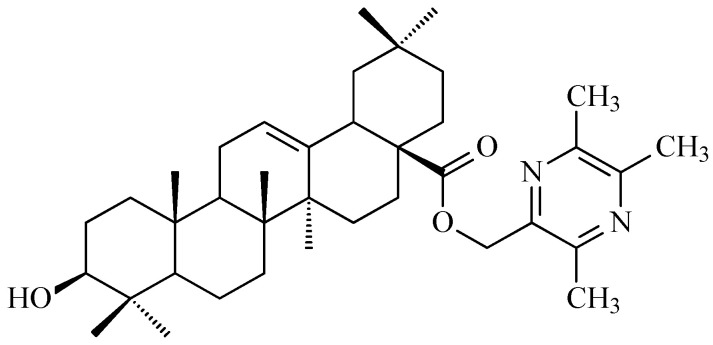
The structure of anticancer agent with 1,3-diazine substituent.

Moreover, derivatives of OA have demonstrated enhanced anticancer activity. Fan et al. investigated an OA derivative (Figure 7; the structure illustrated in the referenced publication appears to exhibit discrepancies when compared with the established structural features of this class of compounds) against human hepatocellular carcinoma SMMC-7721 cells, showing dose- and time-dependent inhibition of proliferation. Treated cells exhibited cytoplasmic shrinkage, membrane blebbing, and apoptotic body formation. The compound decreased BCL-2 levels, increased BAX expression, and activated caspase-9 and caspase-3, indicating initiation of the intrinsic apoptotic pathway. Mitochondrial dysfunction was confirmed by reduced ATP (adenosine triphosphate) levels, loss of mitochondrial membrane potential, and cytochrome c release, highlighting the potential of OA derivatives as candidates for translational research in cancer therapy [99].

OA and its derivatives demonstrate marked anticancer potential, exerting multidirectional effects on signaling pathways involved in apoptosis, autophagy and cellular survival. Their capacity to concurrently suppress proliferative signaling and promote programmed cell death highlights their value as prospective candidates for further exploration in anticancer therapy. Nonetheless, it is important to emphasize that the majority of these observations arise from studies conducted predominantly on cancer cell lines. Therefore, to substantiate these findings, additional research employing more advanced and physiologically relevant models is necessary to more accurately reflect the activity of OA within the complex tumor microenvironment.

### 6.2. Autophagy Regulation, Cell Cycle Arrest, and Anti-Proliferative Effects via PI3K/AKT/mTOR, AKT/JNK Pathways

Oleanolic acid exerts potent antiproliferative and proapoptotic effects in cancer cells by modulating multiple signaling pathways, including the MAPK/ERK axis (extracellular signal-regulated kinases ERK1 and ERK2). In the study by Kim et al., OA induced cell cycle arrest at the G1 or G2 phase depending on the cell type—DU-145 (human prostate cancer), MCF-7 (human breast cancer), or U-87 (human glioblastoma)—which was accompanied by altered expression of cell cycle regulators, including p21, p27, cyclins, and cyclin-dependent kinases (CDKs). Simultaneously, OA activated p53 and increased cytochrome c, BAX, PARP-1, and caspase-3 levels, indicating activation of the mitochondria-dependent apoptotic pathway [100].

These cell cycle and apoptotic effects are consistent with observations in gastric cancer models. In AGS gastric cancer cells, OA inhibited proliferation by inducing apoptosis and autophagy via the PI3K/AKT/mTOR pathway. OA increased BAX and LC3-II (autophagy-related protein light chain 3) expression, while reducing BCL-2 levels and the phosphorylation of PI3K, AKT, and mTOR. In vivo, OA decreased tumor mass without causing organ toxicity, supporting its potential as a natural adjuvant in anticancer therapy [101].

The relevance of these pathways was further confirmed in MKN-28 gastric cancer cells. OA exhibited pronounced proapoptotic activity through activation of the JNK (c-Jun N-terminal kinase) pathway and inhibition of AKT. Treatment reduced cell viability in a dose- and time-dependent manner, induced DNA fragmentation, and caused mitochondrial membrane potential loss. JNK phosphorylation was enhanced, whereas AKT phosphorylation was suppressed, with no detectable effects on ERK or p38. These results indicate that OA triggers mitochondrial apoptosis via modulation of AKT/JNK signaling [102]. Specifically, JNK activation promotes BAX translocation to mitochondria, while AKT inhibition attenuates BCL-2-mediated antiapoptotic signaling, collectively leading to cytochrome c release, apoptosome formation, and caspase-3-mediated cell death.

Beyond apoptosis, OA induces autophagic cell death in hepatocellular carcinoma. In HepG2 and SMMC-7721 cells, OA inhibited the PI3K/AKT/mTOR pathway and increased reactive oxygen species (ROS) generation. Shi et al. demonstrated dose- and time-dependent decreases in cell viability, elevated LC3-II/LC3-I ratios, and enhanced formation of autophagic vacuoles. Pharmacological inhibition of autophagy or ROS scavenging significantly reduced OA-induced cytotoxicity, confirming that both PI3K/AKT/mTOR suppression and ROS accumulation are essential for OA-mediated autophagic cell death [103].

### 6.3. Anti-Angiogenic, Anti-Migration, Anti-Metastatic Properties Linked to Oxidative Microenvironment Modulation

OA exhibits potent anti-angiogenic and anti-migratory effects in colorectal cancer models, as demonstrated by its ability to reduce intratumoral microvessel density and inhibit endothelial cell proliferation, migration, and tube formation. OA suppresses key pro-angiogenic mediators such as VEGF-A and bFGF, which are transcriptionally regulated by oxidative stress-sensitive pathways including STAT3 and SHH. By attenuating STAT3 phosphorylation and downregulating SHH/GLI-1 signaling, OA likely interferes with redox-sensitive transcriptional programs that drive angiogenesis and metastatic potential. These findings suggest that OA’s anti-metastatic efficacy may be partially attributed to its modulation of the oxidative tumor microenvironment, disrupting the signaling cascades that sustain vascularization and cell motility [88]. While the angiogenic response represents a major driver of colorectal tumor progression, OA exhibits broader anticancer functionality across additional tumor models. OA exhibits strong proapoptotic activity in non-small cell lung cancer cells, despite the presence of active multidrug resistance mechanisms and antiapoptotic proteins such as BCL-2 and survivin. Treatment with OA led to caspase-3 activation, DNA fragmentation, and a shift in the BCL-2/BAX balance toward a proapoptotic state. Notably, OA significantly reduced the expression of VEGF, and decreased the number of metastatic foci in the B16-F10 melanoma in vivo mice model. These findings suggest that OA may counteract drug resistance and metastasis by modulating apoptotic and angiogenic pathways [104]. These antimetastatic activities are complemented by direct inhibitory effects on hepatocellular carcinoma growth and invasion. OA shows potent anticancer activity against treatment-resistant hepatocellular carcinoma (Hep-G2) cells by inducing apoptosis, causing cell cycle arrest at the G2/M phase, and significantly inhibiting cell migration and invasion. These effects are associated with suppression of JNK and p38 phosphorylation (p38 is a group of mitogen-activated protein kinases), suggesting OA interferes with key signaling pathways involved in tumor progression [105].

OA demonstrates chemopreventive activity by inducing autophagy in both normal cells and those transformed by the KRAS (Kirsten rat sarcoma virus) oncogene. OA inhibits proliferation, invasiveness, and anchorage-independent growth in MCF-10A (non-tumorigenic human mammary epithelial cell line) and GMC (ganglion mother cells) with active KRAS, and this effect depends on autophagy activation. OA induces autophagy through suppression of the AKT/mTOR/S6K pathway (S6K—ribosomal S6 kinase), a well-known negative regulator of autophagy. Inhibition of autophagy using 3-MA (3-methyladenine; inhibits autophagy via blocking PI3K) abolished the effects of OA, confirming the crucial role of this process in its anticancer activity. These findings indicate that modulation of autophagy-related signaling pathways, particularly AKT/mTOR/S6K, represents a key mechanism underlying the chemopreventive action of OA. Additional pathways, such as oxidative stress responses, may also contribute and warrant further investigation [106].

The nanoformulation of oleanolic acid (OAnano) demonstrated markedly stronger anticancer effects than its conventional form. Due to increased solubility and lipophilicity, OAnano effectively inhibited proliferation, migration, and invasion of U-87 glioma cells both in vitro and in a mouse model. Treatment with OAnano led to activation of the caspase-3 pathway, confirming the induction of apoptosis, while simultaneously reducing the expression of proliferation (Ki-67) and invasion (MMP-7) markers. Changes in Vimentin protein distribution indicated disruption of cytoskeletal organization, further limiting the cells’ migratory capacity. These results suggest that using a nanoliposomal form of OA could represent a promising therapeutic strategy for glioma treatment, combining anticancer efficacy with a limited toxicity profile [107]. A detailed discussion of nanotechnology-based delivery systems, including liposomal formulations, is provided in Section 8.

### 6.4. Cancer Stemness Reduction and Chemosensitization of Oleanolic Acid (e.g., Reversing 5-FU Resistance in Colorectal Models)

Reduction in cancer stemness and enhancement of chemosensitivity represent promising strategies in overcoming drug resistance and tumor recurrence. Cancer stem cells (CSCs), characterized by their capacity for self-renewal, differentiation, and therapy resistance, play a critical role in tumor relapse and poor prognosis. Therapeutic approaches aimed at targeting CSCs have demonstrated that disrupting stemness-associated pathways can resensitize tumors to chemotherapy. For instance, inhibition of the Wnt/β-catenin, Hedgehog, or Notch signaling pathways has been shown to diminish stem-like properties and improve chemosensitivity [108]. Furthermore, CSCs contribute to drug resistance through multiple mechanisms, including epithelial-to-mesenchymal transition (EMT), enhanced DNA repair capacity, quiescence, and high expression of multidrug resistance transporters. EMT not only promotes invasiveness and metastasis but also endows cancer cells with stem-like properties, increasing their survival under therapeutic stress. CSCs often reside in protective niches within the tumor microenvironment, where factors such as hypoxia, inflammation, and cancer-associated fibroblasts further reinforce their resistance to chemotherapy and radiotherapy [109]. Recent advances in lineage-tracing and cell-ablation studies have reshaped our understanding of CSCs, revealing that stemness is not a fixed trait but a dynamic state influenced by the tumor microenvironment. CSCs are no longer viewed as rare, intrinsically quiescent cells; instead, they can be abundant, actively proliferating, and capable of phenotypic interconversion. This plasticity allows non-CSCs to reacquire stem-like properties under specific niche conditions, such as exposure to Wnt, Notch, or EGFR signals, or in response to injury or therapy-induced stress (Wnt, Notch and EGFR are types of cell signaling pathways that are crucial for processes like embryonic development, cell proliferation, differentiation, and migration) [110].

Autophagy, as a degradative and recycling process, plays a key role in maintaining the homeostasis of CSCs. In CSCs, autophagy supports their pluripotency, enables survival under nutrient and oxygen deprivation, regulates migration and invasion, and contributes to resistance against chemotherapy and immunotherapy. Moreover, autophagy can be activated by microenvironmental factors such as hypoxia, further enhancing the adaptive and survival capacity of CSCs. Inhibition of autophagy in various cancer types—including glioma, breast cancer, and leukemia—has been shown to reduce the expression of stemness markers, limit self-renewal ability, and increase treatment sensitivity. Therefore, modulation of autophagy represents a promising therapeutic strategy aimed at eliminating cancer stem-like cells and overcoming treatment resistance [111].

In a study conducted by Ko et al., it was demonstrated that OA inhibits the activity of the enzyme AKR1B10 (aldo-keto reductase family 1 member B10), whose overexpression—induced by arecoline—increases the malignancy of oral squamous cell carcinoma cells (OSCC), promotes their resistance to cisplatin, and enhances cancer stem cell-like properties. OA blocked the TGF-β1/Smad3/Snail signaling pathway responsible for EMT and the development of chemoresistance, leading to reduced expression of stemness (Nanog, Oct4, SOX2) and EMT (Snail, Slug, Twist) markers. As a result, oleanolic acid restored the sensitivity of OSCC cells to cisplatin and limited their ability to proliferate, migrate, and form tumor spheres. The authors suggested that OA may serve as a promising natural adjuvant in oral cancer therapy by reversing chemoresistance and inhibiting the tumorigenic properties of cancer cells [112]. It has also been shown that OA inhibits the growth and self-renewal capacity of colorectal cancer cells, while increasing their sensitivity to the chemotherapeutic agent 5-fluorouracil. This effect results from the blockade of the JAK2/STAT3 (JAK2, Janus kinase 2) signaling pathway, which is responsible for maintaining cancer stem cell-like properties and promoting drug resistance. Consequently, OA reduces the expression of markers such as CD133, Nanog, SOX2, and Oct4, and limits tumor growth in both in vitro and in vivo models. These findings suggest that OA may serve as an effective adjuvant in colorectal cancer therapy, enhancing the efficacy of 5-fluorouracil (5-FU) while potentially reducing its adverse effects [113].

In vitro studies on human breast cancers lines MCF-7 and MDA-MB231 demonstrated that OA significantly reduced the viability of cancer cells while remaining non-toxic to normal breast epithelial cells (MCF-12A). OA increased the levels of autophagy markers, indicating activation of the autophagic pathway. At the same time, no DNA fragmentation or PARP activation was observed, excluding apoptosis as the primary mechanism of cell death. OA inhibited the phosphorylation of AKT and mTOR kinases, key regulators of cancer cell survival, growth, and metabolism, thereby promoting autophagy activation. Additionally, the combination of OA with the PI3K inhibitor exhibited synergistic anticancer effects. These results indicate that OA induces breast cancer cell death primarily through cytotoxic autophagy dependent on the inhibition of the PI3K/AKT/mTOR pathway [114].

OA limits the proliferation, migration, and self-renewal capacity of cancer cells, reduces the expression of stemness and EMT markers, and increases sensitivity to chemotherapeutic agents. These findings suggest that OA may have potential as a natural anticancer adjuvant in in vitro models.

## 7. Enhancing Biological Activity: Structural Modifications

To optimize the bioavailability and therapeutic efficacy of oleanolic acid, intensive research is underway on its chemical modifications and the development of innovative delivery systems. One of the key challenges in the therapeutic use of natural triterpenes, including oleanolic acid, is their low aqueous solubility (4.61 mg/L at 20 °C) and permeability (*P*_app_ = 1.1–1.3 × 10^−6^ cm/s in the apical-to-basolateral direction at 10 and 20 μM), which translate into poor bioavailability (reported absolute F% ≈ 0.7% in rat studies) and rapid metabolism following oral administration [115]. To address this, numerous derivatives and conjugates are being synthesized, demonstrating enhanced biological activity and improved pharmacokinetic properties, which are crucial for their potential clinical application [116]. Furthermore, advanced delivery systems, such as liposomes, polymeric nanoparticles, and cyclodextrin conjugates, are being developed, which can increase solubility, prolong circulation time in the body, and selectively target oleanolic acid delivery to cancer cells while minimizing toxicity to healthy tissues. These approaches aim to improve the therapeutic index of oleanolic acid, which is essential for its translation from laboratory research to clinical practice [117].

Below are presented examples of several types of oleanolic acid derivatives, which serve as excellent illustrations of structural modifications applied to the parent triterpene molecule. Such chemical modifications are aimed at improving pharmacological performance and have led to the development of compounds with enhanced anticancer potential. By introducing substitutions at specific functional groups or by forming hybrid molecules with other bioactive moieties, these derivatives not only demonstrate stronger cytotoxic activity against various tumor cell lines but also often exhibit improved selectivity, reduced toxicity toward normal cells, and better bioavailability. These findings highlight the value of rational structural modification of oleanolic acid as a promising strategy for the design of more effective anticancer agents.

### 7.1. Oleanolic Acid Derivatives with a Modified the C-3 Succinyl or Propionyl Group

**Figure 8:**
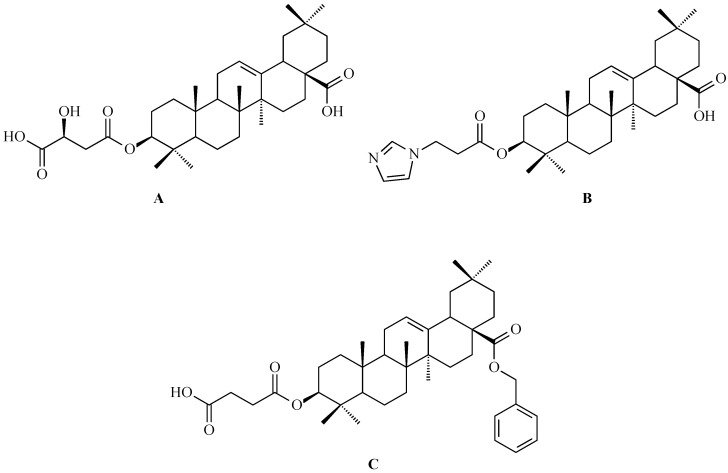
The structures of anticancer agents with modified and unmodified 3-*O*-succinyl moieties.

Hao and colleagues synthesized twelve oleanolic acid derivatives (Figure 8) and tested their cytotoxic activity against prostate (PC-3), breast (MCF-7), lung (A-549), and gastric (BGC-823) cancer cells using 3-(4,5-dimethylthiazol-2-yl)-2,5-diphenyltetrazolium bromide (MTT) assay. Among them, compound with hydroxysuccinic moiety at the C-3 position (compound A, Figure 8) showed the strongest effect on PC-3 cells (IC_50_ = 0.39 μM), while compound with 1H-imidazolepropionyl moiety at the C-3 position (compound B, Figure 8) was most active against A-549 cells (half maximal inhibitory concentration, IC_50_ = 0.22 μM). The structure-activity relationship (SAR) analysis suggested that introducing H-donor substituents at the C-3 position enhances cytotoxicity, particularly against PC-3, A-549, and MCF-7 cells [118].

The 3-*O*-succinyl-28-*O*-benzyl oleanolate derivative (compound C, Figure 8) obtained by Reyes-Zurita and co-workers demonstrated stronger cytotoxic and pro-apoptotic effects than oleanolic acid or its mother compound (benzyl oleanolate). In B16F10 melanoma cells, compound C (Figure 8) inhibited proliferation in a dose-dependent manner, inducing G0/G1 cell-cycle arrest, mitochondrial dysfunction, and extensive apoptosis (72–95%), likely via the intrinsic apoptotic pathway [119].

**Figure 9:**
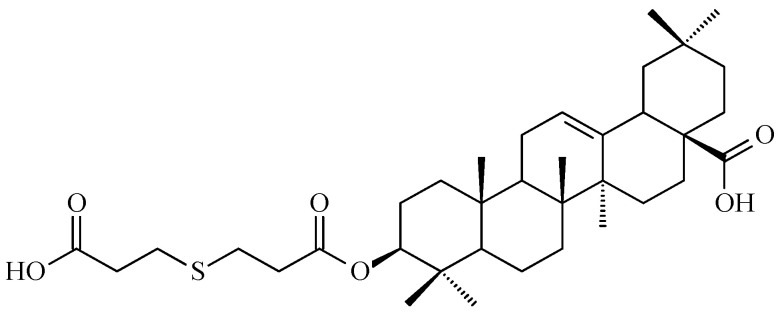
The structure of anticancer agent with thioether moiety.

Li and co-workers synthesized a series of novel oleanolic acid (OA) derivatives containing disulfide, thioether, or selenium ether linkages was and evaluated these compounds for their antiproliferative activity against human liver (BEL-7402, Hep-G2), colon (HCT-116), and normal liver (L02) cell lines using the MTT assay. Among them, compound presented in Figure 9, showed the strongest effect against BEL-7402 cells (IC_50_ = 5.70 ± 0.82 μM). Flow cytometry confirmed that this compound induced G2/M phase arrest leading to apoptosis [120].

### 7.2. Oleanolic Acid Esters

**Figure 10:**
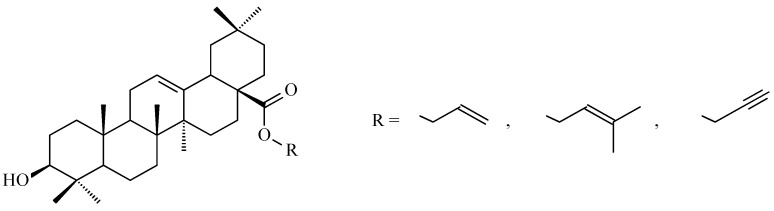
The structures of anticancer agents with unsaturated moieties within ester group.

Mallavadhani and co-workers obtained oleanolic acid esters with unsaturated chain within ester moiety. These esters exhibited superior anticancer activities relative to their parent oleanolic acid when tested against several cancer cell lines, including SiHa and HeLa (cervical cancer cell lines), A-549 (lung cancer), and IMR-32 (neuroblastoma). The unsaturated esters of oleanolic acid (Figure 10) demonstrated particularly promising cytotoxic effects against SiHa, HeLa and A-549 cell lines at a concentration of 10 µM [121].

**Figure 11:**
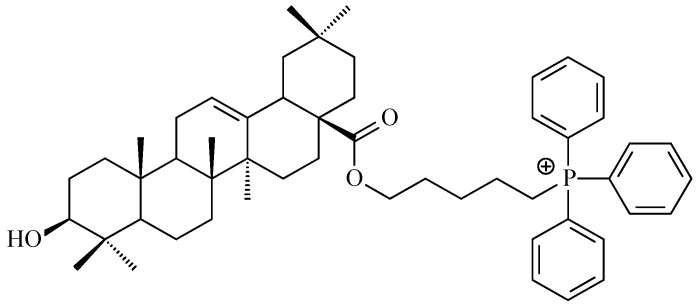
The structure of anticancer agent with triphenylphosphonium cation within ester group.

Li and co-workers proved that introducing a triphenylphosphonium cation to the molecule of modified oleanolic acid structure can enhance the mitochondrial targeting, selectivity, and cytotoxicity of the resulting triterpene. Based on this approach, a series of mitochondria-targeted oleanolic acid derivatives was synthesized and evaluated. Among them, compound with 5-membered chain liked to triphenylphosphonium cation within ester moiety (Figure 11) exhibited potent activity against A-549 lung cancer cells (IC_50_ = 0.81 μM), slightly surpassing doxorubicin (0.97 μM), while showing lower toxicity toward normal cells. Mechanistic investigations revealed that the described new derivative induced apoptosis in a dose-dependent manner, accompanied by ROS generation, mitochondrial membrane depolarization, and upregulation of pro-apoptotic proteins. In addition, the described compound caused G2/M cell cycle arrest, inhibited migration, and showed comparable or superior activity to LY294002, i.e., 2-(4-morpholinyl)-8-phenyl-4H-1-benzopyran-4-one, suggesting involvement of PI3K-AKT pathway inhibition [122].

**Figure 12:**
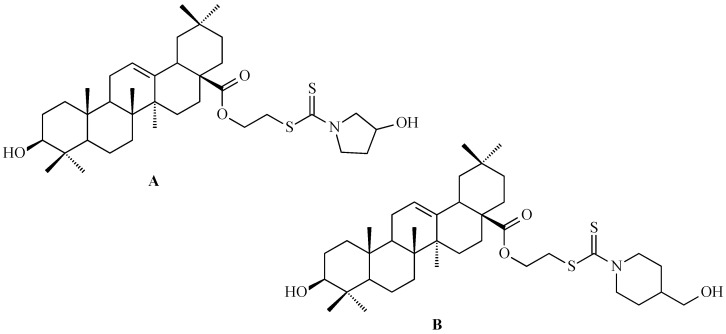
The structures of anticancer agents with modified thiocarbamate moiety within ester group.

Using a pharmacophore hybridization strategy, Tang and co-workers developed a two-step synthesis of novel oleanolic acid-dithiocarbamate conjugates under mild conditions with high yields. Among them, compound A (Figure 12) and compound B (Figure 12) showed the strongest and broad-spectrum antiproliferative activity against Panc-1 (pancreatic carcinoma-1 cell line), A-549, Hep-3B (human hepatocellular carcinoma cell line), Huh-7 (human hepatocellular carcinoma cell line), HT-29 and HeLa cells, with IC_50_ values from 7 to 30 μM for compound A (Figure 12) and from 8 to 50 μM for compound B (Figure 12), while displaying low toxicity to normal cells. These conjugates were up to 30-fold more potent than natural OA based on IC_50_ values [123].

### 7.3. Other Derivatives of Oleanolic Acid Within the C-17 Carboxyl Group

**Figure 13:**
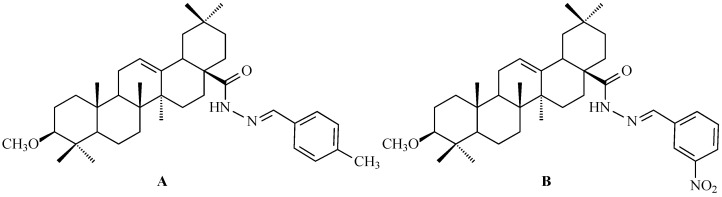
The structures of anticancer agents with modified hydrazide moiety within ester group.

Halil and co-workers synthesized 13 novel oleanolic acid derivatives containing a modified hydrazide moiety within ester group and assessed their cytotoxicity against BEAS-2B (human lung epithelial cell line) and A-549 cell lines. Compounds A and B, presented in Figure 13, with IC_50_ = 2.96 µM and 2.53 µM, respectively, showed the lowest toxicity toward BEAS-2B cells, while exhibiting strong activity against A-549 cells (IC_50_ = 0.08 and 0.22 µM, respectively). Notably, first of the mentioned compound (compound A, Figure 13) demonstrated cytotoxicity comparable to doxorubicin in A-549 cells (IC_50_ = 0.14 µM) [124].

**Figure 14:**
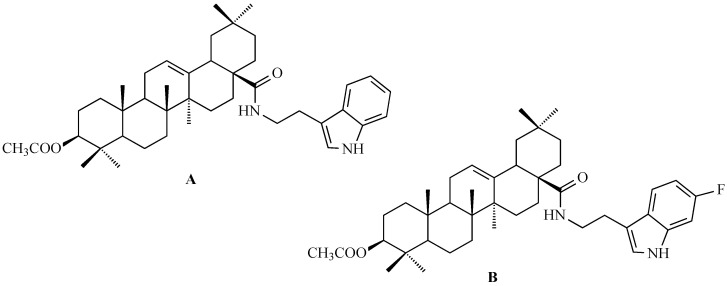
The structures of anticancer agents with tryptamine or fluorotryptamine moiety within ester group.

Bildziukevich and co-workers synthesized tryptamine and fluorotryptamine hybrids of oleanolic acid and evaluated their anticancer activity against CEM (human T-cell leukemia cell line), MCF-7, HeLa, and G-361 (human melanoma cell line) cancers. The tryptamine amide (compound A, Figure 14) showed notable cytotoxicity in HeLa (IC_50_ = 8.7 µM) and G-361 (IC_50_ = 9.0 µM) cells. Fluorotryptamine derivative (compound B, Figure 14) were active in HeLa cells (IC_50_ = 6.7 µM). Both compounds showed a strong induction of apoptosis in HeLa and G-361 cells after 24 h [125].

**Figure 15:**
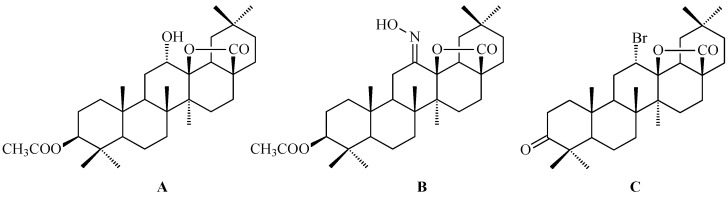
The structures of anticancer agents with lactone system.

Bednarczyk-Cwynar and co-workers presented a series of oleanolic acid derivatives, including lactones and bromolactones, which were tested for cytotoxic, antioxidant, and pharmacokinetic properties. Molecular docking against the EGFR (epidermal growth factor receptor) tyrosine kinase domain indicated 12-hydroxyiminolactone (compound B, Figure 15) as the most promising candidate, showing strong interactions with key residues (LYS 721, lysine located at position 721 in the protein and ASP 831, aspartic acid located at position 831 in the protein). The 12-hydroxylactone (compound A, Figure 15), hydroxyiminolactone (compound B, Figure 15) and 3-oxo-12-bromolactone (compound C, Figure 15) displayed micromolar IC_50_ values (from ~2 to ~10 µM) with favorable selectivity. ADMETox evaluation (absorption, distribution, metabolism, excretion, and toxicity) supported good pharmacokinetic and safety profiles. Overall, the mentioned compounds emerged as leading candidates for further development of EGFR-targeted anticancer agents, though clinical translation of oleanolic acid derivatives remains limited by bioavailability and metabolic stability [126].

### 7.4. Oleanolic Acid Acylated Oximes

**Figure 16:**
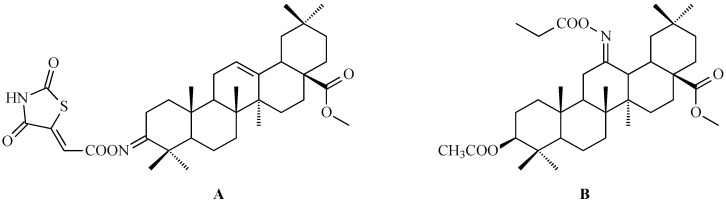
The structures of anticancer agents with modified A or C ring within oleanane structure.

Kaminskyy and colleagues reported the synthesis and biological evaluation of novel acylated oxime derivatives of oleanolic acid bearing 4-thiazolidinone-3(5)-carboxylic acid fragments. The anticancer potential of these compounds was assessed in vitro by the National Cancer Institute (NCI), and SAR were analyzed. Among the synthesized derivatives, the methyl ester of oleanolic acid oxime featuring a (2,4-thiazolidinedione-5-ylidene)-carboxyimino group at the C-3 position (compound A, Figure 16) exhibited the highest activity, with mean pGI_50_ = 5.51/5.57, pTGI = 5.09/5.13, and pLC_50_ = 4.62/4.64 values (pGI_50_, negative logarithm of the half-maximal inhibitory concentration; pTGI, negative logarithm of the total inhibition of cell growth; pLC_50_, negative logarithm of the half-maximal lethal concentration). This compound demonstrated low toxicity and moderate efficacy in the in vivo hollow fiber assay, outperforming related analogs [127].

Bednarczyk-Cwynar and co-workers synthesized a series of novel methyl oleanolate derivatives featuring structural modifications within the A- and/or C-rings, specifically involving alterations of the hydroxyimino group at the C-3 or the C-12 positions. Most of the obtained derivatives exhibited potent cytotoxic effects against KB (human epidermal oral carcinoma cell line), MCF-7, and HeLa cancer cell lines, showing markedly higher activity compared to the parent oleanolic acid. Among these compounds, derivatives bearing an acyloxyimino substituent demonstrated the strongest anticancer potential, with the propionoxyimino derivative at the C-12 position (compound B, Figure 16) identified as the most active. This compound displayed IC_50_ values ranging from 0.72 to 2.13 μM across the tested cell lines, highlighting the significance of targeted structural modification in enhancing the antitumor efficacy of oleanolic acid analogs [128].

**Figure 17:**
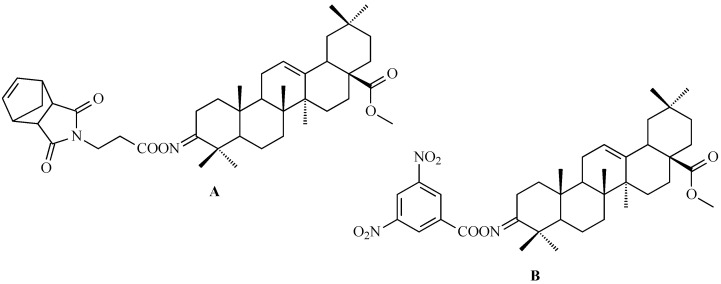
The structures of anticancer agents with norbornene or 3,5-dinitro moiety within acyloxyimino function at the C-3 position.

In 2017, Bednarczyk-Cwynar and colleagues reported the synthesis of a novel series of acylated oxime derivatives of oleanolic acid. These hybrid molecules were designed by linking oleanolic acid oximes with carboxylic acids containing short alkyl chains attached to nitrogen atoms of norbornene-2,3-dicarboximide moieties, resulting in structurally diverse conjugates. The newly obtained compounds were thoroughly evaluated for their cytotoxic potential using the MTT assay on several human cancer cell lines, including HeLa, KB, MCF-7, and Hep-G2, as well as normal human dermal fibroblasts (HDF), with unmodified oleanolic acid serving as the reference compound. The results demonstrated that most derivatives exhibited significantly enhanced antiproliferative activity compared to the parent molecule. Among them, the derivative with a propionoxyimino linker joining norbornene and oleanane skeletons (compound A, Figure 17) showed the most pronounced cytotoxic effects, with IC_50_ values ranging from 2.75 μM for MCF-7 cells to 4.36 μM for HDF cells. These findings highlight that structural modifications involving norbornene-imide moieties can substantially improve the biological activity of oleanolic acid, suggesting that such hybridization strategies represent a promising direction for the development of more potent and selective anticancer agents [129].

In a subsequent study, Bednarczyk-Cwynar and co-workers reported the synthesis of another highly effective anticancer agent derived from oleanolic acid, designed as an acylated oxime. In this work, the researchers obtained a methyl oleanonate oxime acylated with 3,5-dinitrobenzoic acid (compound B, Figure 17), which demonstrated significant cytotoxic potential. The compound was evaluated for its anticancer activity against a panel of human cancer cell lines, including HeLa, KB, MCF-7, and A-549, showing IC_50_ values of approximately 5 µM, indicating potent inhibitory effects comparable to or stronger than those of the parent compound [130].

Furthermore, the synthesized oxime derivative was subjected to electroreduction studies, which provided valuable insights into its redox properties and possible mechanisms of cytotoxic action. The authors emphasized that the application of electrochemical techniques as preclinical screening tools offers a powerful and advantageous approach for the early identification of compounds with potential cytotoxic or anticancer effects, complementing traditional biological assays and contributing to more efficient drug discovery workflows [130].

As a continuation of their research on the anticancer activity of oleanolic acid acylated oximes, Bednarczyk-Cwynar and Ruszkowski published a study describing the synthesis and biological evaluation of three series of novel derivatives [131]:

(i) Methyl oleanonate oxime derivatives;
(ii) Methyl oleanonate oxime derivatives bearing an additional 11-oxo function;
(iii) Morpholide derivatives of oleanonic acid oxime.

In these compounds, the oxime groups were acylated with aliphatic or aromatic carboxylic acids, generating a diverse set of structures. The newly synthesized derivatives were comprehensively evaluated for ADMETox properties and cytotoxic activity against several human cancer cell lines, including HeLa, KB, MCF-7, A-549, and HDF, using the MTT assay.

**Figure 18:**
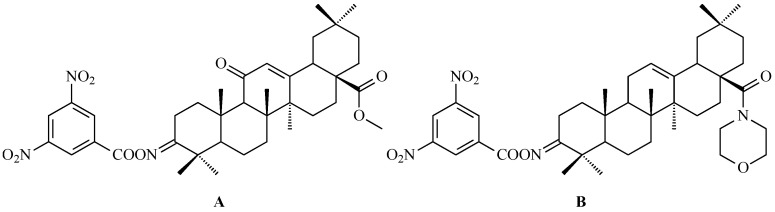
The structures of anticancer agents with 3,5-dinitrobenzoic moiety within acyloxyimino function at the C-3 position.

The results revealed that several of the acylated oximes, particularly those bearing aromatic rings substituted with two nitro groups (e.g., compounds A and B, Figure 18), displayed remarkably high cytotoxic potency, with IC_50_ values in the micromolar range. These derivatives significantly inhibited the proliferation of all tested cancer cell lines while maintaining a favorable selectivity index (SI). The authors noticed that the introduction of electron-withdrawing groups such as 3,5-dinitro moieties markedly enhanced the anticancer potential of oleanolic acid oxime derivatives, yielding promising candidates with potent cytotoxic effects and satisfactory pharmacokinetic and safety profiles, as indicated by the ADMETox analysis [131].

### 7.5. Oleanolic Acid Lactams

**Figure 19:**
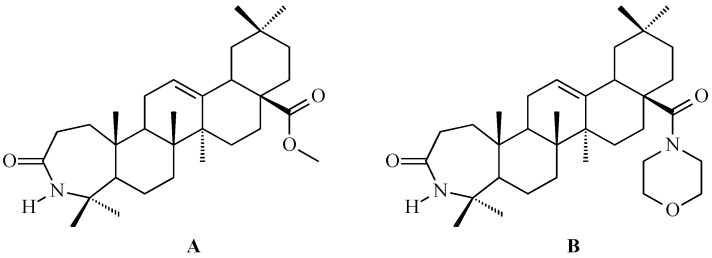
The structures of anticancer agents with A-lactam system.

Bednarczyk-Cwynar and co-workers presented a synthesis of a series of oleanolic acid derivatives—including ketones, oximes, lactams, and nitriles. These semisynthetic compounds exhibited diverse functional modifications within the oleanane scaffold: the C-3 position contained oxo, hydroxyimino, lactam, or nitrile groups; the C-17 position carried either an esterified or unmodified carboxyl moiety; and, in selected cases, an additional oxo group was introduced at the C-11 position. Such structural variations were designed to investigate the influence of these substituents on biological activity. The cytotoxic potential of all derivatives was evaluated using the MTT assay against four human cancer cell lines: HeLa, KB, MCF-7, and Hep-G2. The results revealed that several oxime derivatives and, notably, all the lactam analogs (e.g., compounds A and B, Figure 19) demonstrated pronounced cytotoxic effects, with IC_50_ in the low micromolar range (~1.5–~4.0 µM). These findings indicate that the introduction of hydroxyimino or lactam functionalities at the C-3 position significantly enhances the anticancer potential of oleanolic acid, likely by altering its interaction with cellular targets or improving membrane permeability [132].

### 7.6. Oleanolic Acid Hybrids of Various Type

**Figure 20:**
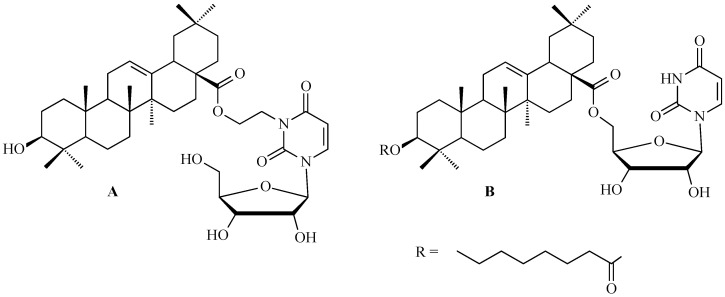
The structures of anticancer agents with uridine-sugar moiety within ester group.

Through pharmacophore hybridization, Cheng and his group synthesized ten uridine-oleanolic acid hybrids, most of which showed strong antiproliferative activity against Hep-G2, A-549, BGC-823, MCF-7, and PC-3 cell lines (IC_50_ < 8 μM, sometimes IC_50_ < 0.1 μM). Hybrids presented in Figure 20 (compounds A and B) displayed low toxicity towards normal liver cells (HL-7702). Mechanistic studies demonstrated that hybrid A (Figure 20) induced apoptosis in Hep-G2 cells by disrupting mitochondrial membrane potential, causing G1-phase arrest, and activating caspase-3/9 [133].

**Figure 21:**
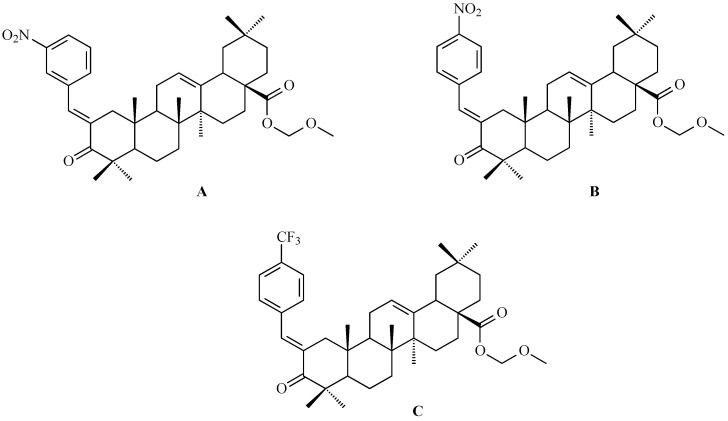
The structures of anticancer agents with α-unsaturated ketone moieties.

Şenol and co-workers synthesized a series of α,β-unsaturated ketone derivatives of oleanolic acid and evaluated anticancer activity of these compounds against human prostate cancer PC-3 cells. Compounds presented in Figure 21 (compounds A, B and C) showed the strongest cytotoxicity (IC_50_ values of 7.8, 8.9, and 8.8 μM, respectively) with lower toxicity toward normal HUVEC (human umbilical vein endothelial cells) compared to doxorubicin. ADME profiling confirmed favorable drug-likeness, while docking and MM-GBSA (molecular mechanics/generalized born surface area) analyses indicated strong and stable interactions with PARP1, PI3K, and mTOR. Molecular dynamics simulations further supported the stability of these complexes under physiological conditions [134].

### 7.7. CDDO-Me

**Figure 22:**
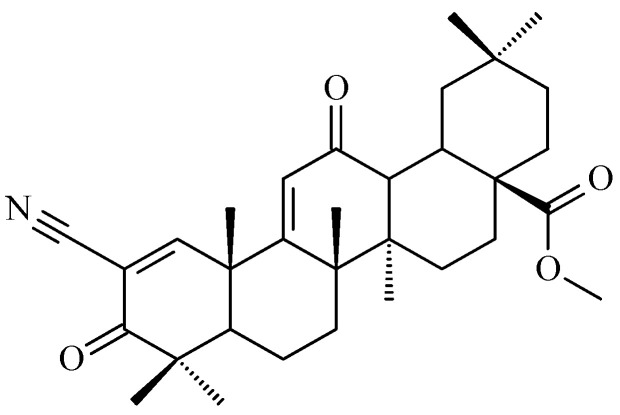
The structure of anticancer agent Bardoxolone-methyl (known also as CDDO-Me).

The semisynthetic triterpenoid methyl 2-cyano-3,12-dioxooleana-1,9(11)-dien-28-oic acid methyl ester (commonly referred to as CDDO-Me or Bardoxolone methyl) (Figure 22) is one of the most potent and extensively studied synthetic derivatives of oleanolic acid. It was designed to enhance the pharmacological potential of natural oleanane-type triterpenes through structural modifications that increase both cytotoxic potency and bioavailability. The synthesis of CDDO-Me was first described by Honda et al., who reported the preparation of a series of oleanane derivatives containing modifications at the C-2, the C-3, and the C-12 positions, resulting in dioxo and cyano functional groups that strongly influence biological activity. These derivatives, including CDDO and its methyl ester CDDO-Me, showed pronounced inhibitory effects on nitric oxide production in activated macrophages, indicating potential anti-inflammatory and anticancer properties [135].

Subsequent landmark studies by Suh et al. revealed that both CDDO and CDDO-Me (Bardoxolone and its methyl ester, respectively) exhibit exceptionally high anticancer potential, selectively inducing apoptosis and inhibiting proliferation in a variety of human cancer cell lines, including breast carcinoma (MCF-7), prostate carcinoma (LNCaP), colon adenocarcinoma (HT-29), and leukemia (HL-60) cells. Importantly, CDDO-Me was shown to act through multiple molecular mechanisms, including activation of pro-apoptotic signaling, inhibition of anti-apoptotic proteins such as BCL-2 and BCL-xL, and induction of caspase-dependent apoptosis [136].

In the following years, the compound attracted wide attention as a multi-target anticancer agent, capable of modulating transcription factors and signaling pathways that play crucial roles in cancer progression. Studies conducted by Liby, Sporn, and colleagues demonstrated that CDDO-Me effectively inhibits the NF-κB and STAT3 signaling pathways—key regulators of inflammation, proliferation, and survival in tumor cells—while simultaneously activating the Nrf2/Keap1 pathway, which enhances cellular antioxidant defense [137].

CDDO-Me has demonstrated cytotoxic effects across a broad range of cancer models, including, e.g., U-937 leukemia cells, B-16 murine melanoma and L-1210 murine leukemia cells [138], MCF-7 breast cancer and MDA-MB-435 melanoma cells [139], 2774, SKOV-3, CAOV-3, OVCAR-3, NMP-1, HEY, 2008 and 2008.C-13 epithelial ovarian carcinoma cells [140], and many others. Remarkably, CDDO-Me was effective at low micromolar and submicromolar concentrations, which underscores its potency compared to the parent oleanolic acid. Its mechanism of action extends beyond direct tumor cytotoxicity—it also involves anti-inflammatory and antioxidant effects, suppression of ROS generation, and modulation of gene expression through redox-sensitive transcription factors.

Moreover, CDDO-Me has been shown to act as a Nrf2 activator, inducing phase II detoxifying and antioxidant enzymes such as HO-1 and NQO1 [141]. The modulation of these pathways links CDDO-Me’s anticancer efficacy with its anti-inflammatory and cytoprotective potential, making it a unique dual-function compound.

Due to its high potency and multi-target activity, CDDO-Me progressed to clinical development, primarily for treating chronic kidney disease and cancer-related inflammation. However, despite promising early-phase results, later-stage clinical trials revealed dose-dependent toxicity, particularly cardiovascular side effects, which prompted further SAR studies to design safer analogs [142].

In summary, CDDO-Me remains a milestone compound in triterpenoid chemistry, serving as a prototype for designing multifunctional oleanane derivatives with improved anticancer, anti-inflammatory, and antioxidant properties. Its discovery marked a turning point in the medicinal chemistry of triterpenes, demonstrating that rational structural modifications of natural molecules can yield highly potent and therapeutically relevant semisynthetic analogs.

## 8. Enhancing Biological Activity: Nanotechnology Techniques Improving Solubility and Bioavailability

The use of nanotechnology, including encapsulation of oleanolic acid in liposomes, polymer nanoparticles, micelles, or conjugates with cyclodextrins, is a promising strategy for improving its solubility, stability, and bioavailability, and thus its therapeutic efficacy. These advanced delivery systems enable precise targeting of oleanolic acid to cancer cells while minimizing its toxicity to healthy tissues. The use of nanocarriers allows for bypassing biological barriers such as enzymatic degradation or low permeability of cell membranes, which is crucial for compounds with limited solubility in an aqueous environment. Furthermore, the use of nanotechnologies can increase the bioavailability of oleanolic acid through controlled drug release, ensuring consistent therapeutic concentrations in target tissues [143].

### 8.1. Liposomes

Liposomes as lipid vesicles, are excellent carriers for lipophilic compounds such as oleanolic acid, enabling their effective delivery to target tissues and protecting them from degradation in the body. Furthermore, the ability to modify the liposome surface by attaching ligands specific to receptors overexpressed on cancer cells allows for targeted delivery of OA, increasing its selectivity and minimizing systemic toxicity. Therefore, the use of liposomes opens the door to the development of precise and more effective OA-based therapies, which could revolutionize the approach to cancer treatment [144].

This advantage is particularly important for compounds with limited water solubility, such as pentacyclic triterpenes, for which nanocarrier systems are key to improving pharmacological efficacy. This approach enables not only increased bioavailability but also control over drug release at the target site, which translates into better therapeutic outcomes and reduced doses required to achieve effect [145]. Furthermore, nanocarriers can also bypass multidrug resistance mechanisms by transporting active compounds directly into cancer cells, which is a significant advantage in the fight against advanced cancers. Nanomaterials, including nanoliposomes, have the ability to effectively deliver poorly soluble drugs to tumor sites, which is crucial for increasing their penetration through blood circulation barriers and for controlled release [144].

As an example, Luo et al. developed oleanolic acid-encapsulated multivesicular liposomes (OA-MVLs) using a double-emulsion method, optimized via central composite design. The resulting spherical particles had an average size of approximately 11.6 μm and an encapsulation efficiency of 82.3%. OA-MVLs exhibited a sustained-release profile in vitro, consistent with the Ritger–Peppas model, and significantly inhibited the proliferation of Hep-G2 cells, as confirmed by MTT assays and fluorescence microscopy. In vivo, OA-MVLs demonstrated prolonged circulation time and reduced toxicity compared to the free OA solution, effectively suppressing H-22 hepatoma growth and extending survival in tumor-bearing mice. These findings indicate that OA-MVLs represent a promising controlled-release formulation for improving the therapeutic efficacy of oleanolic acid in cancer treatment [145].

### 8.2. Proliposomes

The concept of proliposomes was developed as an advanced formulation approach to enhance the stability and shelf life of conventional liposomes, which are often limited by issues such as aggregation, fusion, and leakage of the encapsulated drug during storage. Proliposomes are dry, free-flowing particulate systems composed of phospholipids and other lipophilic components that can be rapidly hydrated upon contact with aqueous media to form uniform liposomal dispersions. This transformation occurs spontaneously, resulting in the in situ generation of liposomes with properties comparable to freshly prepared formulations [146].

By maintaining the lipids in a dry and stable state until reconstitution, proliposomes overcome the physicochemical instability associated with liquid liposomal formulations. Moreover, they offer additional advantages such as improved reproducibility, ease of transportation, enhanced bioavailability of poorly soluble compounds, and suitability for oral, parenteral, or topical delivery. Owing to these benefits, proliposomes have emerged as a promising intermediate in modern drug delivery systems, bridging the gap between traditional liposomes and more complex nanocarrier technologies [146].

Wang and Gao proposed a novel method for the preparation of oleanolic acid proliposomes, which were subsequently converted into liposomes for further evaluation. The physicochemical properties of the resulting liposomes, including surface morphology, particle size, entrapment efficiency, and in vitro absorption, were systematically investigated. The results demonstrated that oleanolic acid was successfully encapsulated within the liposomal structure. The obtained liposomes exhibited a small and uniform particle size and a high entrapment efficiency of 85.65 ± 7.96%. It was further observed that both the pH of the liposomal suspension and the phospholipid-to-drug ratio (P/D) significantly influenced encapsulation efficiency: higher pH values and an increased P/D ratio (from 5:1 to 10:1) led to improved drug loading. Moreover, in vitro intestinal absorption studies revealed that the liposomal formulation significantly enhanced the absorption of oleanolic acid compared to the control group. The study therefore confirmed that this new proliposome-based method enables efficient encapsulation and improved absorption of OA, representing an important step toward the development of optimized liposomal delivery systems for this compound [147].

### 8.3. Nanoliposomes

Nanoliposomes are liposomes with nanometric dimensions, typically below 200 nm in diameter, often ranging between 50 and 150 nm. Due to their small size, they possess a larger surface area-to-volume ratio, which enhances their interaction with biological membranes, improves bioavailability and cellular penetration, and prolongs circulation time in the body—particularly when stabilized, for example, by PEGylation (PEGylation, the process of both covalent and non-covalent attachment or amalgamation of polyethylene glycol, PEG). Nanoliposomes are frequently employed in targeted drug delivery, especially for anticancer agents or drugs with poor aqueous solubility [148].

An illustrative example of research focused on improving the bioavailability of oleanolic acid through nanotechnology is the study conducted by Tang et al. In their work, the authors developed and optimized PEGylated oleanolic acid nanoliposomes designed to enhance the solubility, stability, and pharmacokinetic properties of the compound These nanoliposomal formulations demonstrated superior bioavailability and therapeutic efficacy (on the U-14 cervical carcinoma cell line) compared to free oleanolic acid, primarily due to their ability to prolong systemic circulation, enhance tumor accumulation, and reduce off-target toxicity. Moreover, the encapsulated oleanolic acid exhibited stronger antitumor activity and lower cytotoxicity toward healthy tissues, highlighting the advantages of nanoliposomal delivery in targeted cancer therapy. This study provides valuable insight into the potential of nanocarrier-based systems as a promising approach for the efficient delivery of poorly soluble triterpenoids such as oleanolic acid [149].

### 8.4. Nanoparticles

Nanoparticles have been developed as an effective strategy to address the compound’s inherently poor aqueous solubility and limited bioavailability. These nanosystems are typically fabricated via self-assembly techniques or through conjugation with biocompatible materials such as polymers, lipids, or proteins. The resulting nanoparticles serve as advanced drug delivery carriers, capable of enhancing the stability, solubility, and targeted delivery of oleanolic acid and other therapeutic agents [150]. Recent studies have demonstrated that oleanolic acid nanoparticles exhibit notable potential in cancer therapy, protection against gastrointestinal injury, and antioxidant activity, underscoring their versatility in biomedical applications.

Man et al. developed OA-loaded nanoparticles based on mPEG-PLGA (methoxy poly(ethylene) glycol and poly(lactic-*co*-glycolic acid) poliester) and mPEG-PLA (methoxy poly(ethylene) glycol and poly(lactic acid) poliester) copolymers to enhance the delivery and therapeutic efficacy of OA. The nanoparticles were obtained using the nanoprecipitation method and thoroughly characterized in terms of their physicochemical properties. The resulting formulations demonstrated encapsulation efficiencies ranging from 40% to 75% and exhibited particle sizes of approximately 200–250 nm, which is optimal for tumor targeting via the enhanced permeability and retention (EPR) effect [151].

The nanoparticles possessed a negative surface charge, contributing to their excellent colloidal stability, with no signs of aggregation observed for over 20 weeks. In vitro studies confirmed that the OA-loaded nanoparticles induced significant cytotoxic and pro-apoptotic effects in various cancer cell lines. Although both mPEG-PLGA and mPEG-PLA formulations shared comparable physicochemical characteristics, the OA-loaded mPEG-P(D,L)LGA (methoxy poly(ethylene) glycol and poly(D,L-lactic-*co*-glycolic acid) poliester) nanoparticles demonstrated superior cytotoxicity toward tumor cells, suggesting a higher efficiency in OA delivery and therapeutic performance [151].

### 8.5. Nanocapsules

Taking into consideration their internal architecture, polymeric nanoparticles are generally classified as nanospheres or nanocapsules. Polymeric nanospheres are solid particles composed of a uniform polymeric matrix, whereas nanocapsules possess a core–shell structure, featuring a liquid or solid core surrounded by a polymeric membrane. Owing to this configuration, nanocapsules have gained significant attention in recent years for drug delivery applications. Their core–shell design enhances drug-loading capacity and minimizes polymer content compared with nanospheres. Moreover, the polymeric shell protects the encapsulated drug from degradation or burst release caused by pH, temperature, or enzymatic factors, while also allowing surface functionalization for targeted delivery [152].

Huang and co-workers employed a dynamic penetration system to evaluate the sustained-release behavior of oleanolic acid from the nanocapsule formulation. The release profile, analyzed by HPLC (high-performance liquid chromatography), was found to fit the Weibull kinetic model, indicating a controlled and predictable drug-release pattern. The results demonstrated that the OA-loaded nanocapsules provided a significantly prolonged release compared to the control formulation, exhibiting a half-release time (t_1/2_) approximately 6.7 times longer than that of the free OA. This sustained-release property suggests that the nanocapsule delivery system effectively enhances the bioavailability and therapeutic duration of OA, reducing the frequency of administration and improving its pharmacological performance [153].

### 8.6. Solid Dispersions

A solid dispersion (SD) is a system in which one or more active ingredients are dispersed in an inert carrier in the solid state, typically prepared using fusion, solvent, or solvent-fusion techniques. In such systems, the reduction in drug particle size and improvement in wettability and dispersibility significantly enhance dissolution and bioavailability, particularly for hydrophobic compounds. Hydrophilic polymers, such as polyvinylpyrrolidone (PVP), are commonly used carriers due to their high solubility, biocompatibility, and low toxicity. Recently, incorporating surface-active agents into solid dispersions has gained attention, offering further improvement in drug dissolution efficiency [154]. Goldberg et al. demonstrated that in solid dispersions, not all of the drug necessarily exists in a microcrystalline form—a portion may be molecularly dispersed within the carrier matrix, forming a solid solution [155].

Liu and Wang conducted a study aimed at improving the dissolution behavior of oleanolic acid through the development of solid dispersion systems composed of the drug, a polymeric carrier, and a surfactant [154]. For comparison, binary solid dispersions containing OA and PVP were prepared, while polysorbate 80, a nonionic surfactant, was introduced as a third component to obtain ternary solid dispersions. The resulting formulations were characterized using differential scanning calorimetry (DSC), Fourier-transform infrared (FTIR) spectroscopy, and dissolution testing. The analyses revealed that crystallization of OA was effectively suppressed in the solid dispersions. Both binary and ternary systems significantly enhanced the dissolution rate of OA compared to the pure drug, with the ternary dispersions showing superior performance. The inclusion of polysorbate 80 was found to play a crucial role in further improving the dissolution efficiency of the formulations [154].

### 8.7. Complexes with Cyclodextrins

Cyclodextrins have gained great importance in the pharmaceutical industry due to their ability to enhance the solubility, stability, and bioavailability of poorly soluble drugs. These cyclic oligosaccharides, composed of α-1,4-linked glucose units derived from the enzymatic degradation of starch, possess a unique structure with a hydrophilic exterior and a hydrophobic cavity. This allows them to form inclusion complexes with hydrophobic molecules, significantly improving their aqueous solubility and stability. Given that up to 40% of marketed drugs and 90% of those under development suffer from poor water solubility, cyclodextrins are increasingly used in pharmaceuticals, cosmetics, biotechnology, and nanomedicine. Recent studies highlight their roles in improving drug efficacy, reducing toxicity, and enabling controlled release systems, often enhanced further by incorporating polymers, amino acids, or hyaluronic acid [156].

Pentacyclic triterpenes such as oleanolic acid (OA) and ursolic acid (UA) can form inclusion complexes with cyclodextrins, enhancing their aqueous solubility and pharmacological activity. In their study, Stelling-Ferez and co-workers selected 2-hydroxypropyl-β-cyclodextrin and 2-hydroxypropyl-γ-cyclodextrin as host molecules based on virtual screening results. The formation of inclusion complexes was confirmed using scanning electron microscopy, differential scanning calorimetry, and X-ray diffraction. In vitro assays on melanoma cell lines showed that the complexes exhibited stronger antiproliferative effects than the pure compounds, particularly for ursolic acid, with the 2-hydroxypropyl-γ-cyclodextrin complex showing the highest activity. These findings indicate that complexation with hydrophilic cyclodextrins is an effective strategy to enhance the bioactivity of triterpene acids [157].

### 8.8. SMEDDS

The self-microemulsifying drug delivery system (SMEDDS) has emerged as an important approach for enhancing the bioavailability of poorly water-soluble compounds. Nevertheless, several limitations are associated with SMEDDS formulations, including in vivo drug precipitation, challenges in formulation handling, limited lymphatic uptake, absence of reliable predictive in vitro models, and the susceptibility of unsaturated fatty acids to oxidation. These drawbacks constrain the broader application of SMEDDS [158].

The incorporation of polymers or precipitation inhibitors into lipid-based formulations can help sustain drug supersaturation following dispersion, thereby enhancing bioavailability and reducing variability in systemic exposure. Furthermore, the development of solid SMEDDS offers a solution to the handling and stability issues typically encountered with liquid formulations. Additionally, the use of medium-chain triglycerides (MCTs) and appropriate antioxidants can effectively mitigate the oxidation of unsaturated fatty acids, thus addressing some of the key challenges associated with SMEDDS [158].

Yang et al. developed SMEDDS to enhance the solubility and oral bioavailability of oleanolic acid. The formulation was optimized through solubility studies, compatibility assessments, and construction of pseudoternary phase diagrams. Comprehensive characterization of the oleanolic acid-loaded SMEDDS was performed, including evaluation of its morphology, droplet size distribution, zeta potential, viscosity, electrical conductivity, and refractive index [159].

The results demonstrated that, compared with an oleanolic acid solution, the SMEDDS formulation enabled a sustained in vitro release profile of the drug. A highly selective and sensitive high-performance liquid chromatography-mass spectrometry (HPLC-MS) method was employed to quantify oleanolic acid concentrations in rat plasma. This analytical technique was subsequently applied in a pharmacokinetic comparison between the oleanolic acid-loaded SMEDDS and a conventional tablet formulation in rats. According to the authors, the SMEDDS achieved a remarkable 5.07-fold enhancement in the oral bioavailability of oleanolic acid relative to the marketed tablet formulation. These findings highlight the promising potential of SMEDDS as an effective strategy for the oral delivery of oleanolic acid [159].

### 8.9. Nanoemulsions

Nanoemulsions, characterized by small droplet sizes (20–200 nm), offer a large surface area, which promotes faster dissolution and absorption of oleanolic acid, increasing its bioavailability and stability under physiological conditions. Their use is particularly beneficial for lipophilic compounds, enabling more efficient delivery to target sites and minimizing degradation, which translates into increased therapeutic efficacy. Additionally, nanoemulsions can provide protection against enzymatic degradation in the gastrointestinal tract, which is important for maintaining the integrity and activity of oleanolic acid, especially during oral administration [160].

Alvarado et al. designed and optimized oil-in-water nanoemulsions for dermal delivery of natural and synthetic pentacyclic triterpenes mixtures with known anti-inflammatory properties. Using pseudo-ternary phase diagrams, the formulations were developed with castor oil (oil phase), Labrasol (surfactant), Transcutol-P (co-surfactant), and propylene glycol (aqueous phase). Two nanoemulsions with droplet sizes below 600 nm were obtained, showing viscosities of 51.97 ± 4.57 mPa × s (natural triterpenes) and 55.33 ± 0.28 mPa × s (synthetic triterpenes). In vivo tests confirmed that both nanoemulsions were non-toxic and non-irritant. Notably, the formulation containing natural triterpenes exhibited superior anti-inflammatory activity, demonstrating the potential of o/w (oil/water) nanoemulsions for effective dermal delivery of pentacyclic triterpenes [160].

In another study, Alvarado et al. investigated the antioxidant and anticancer potential of OA and UA extracted from *Plumeria obtusa*, both in free form and encapsulated within a nanoemulsion (NEm) system, using the B-16 murine melanoma cell line. The nanoemulsion was characterized by dynamic light scattering, transmission electron microscopy, and viscosity analysis. Antioxidant activity was assessed via DPPH (2,2-diphenyl-1-picrylhydrazyl) radical scavenging, while cytotoxicity was evaluated using the sulforhodamine-B assay. The OA/UA natural mixture demonstrated strong antioxidant activity, with over 85% DPPH inhibition both with and without UV (ultraviolet) irradiation. When incorporated into the NEm system, comparable antioxidant effects were maintained, and cytotoxic activity was enhanced, with an IC_50_ of 2.9 µM compared to 17.4 µM for the free compounds. These results highlight the nanoemulsion’s ability to potentiate the biological effects of OA and UA [161].

### 8.10. Micelles

Polymeric micelles can effectively solubilize oleanolic acid, improving its aqueous solubility and stability under physiological conditions, which increases its bioavailability and ability to reach cancer cells. They are characterized by a core-shell structure, which ensures kinetic stability and effective solubilization of hydrophobic substances, which is crucial for increasing the therapeutic potential of numerous substances. Furthermore, the small size of polymeric micelles allows for easy administration via various routes, including intravenous, oral, or transdermal, expanding their potential applications in personalized medicine [162].

An et al. developed a polymeric micelle formulation to improve the solubility and cosmetic applicability of oleanolic acid as an active ingredient for wrinkle reduction. Solubility was assessed in various solubilizers, surfactants, and polymers, while micelle characteristics were analyzed using electrophoretic light scattering and cryo-scanning electron microscopy (cryo-SEM). Encapsulation efficiency, stability (40 °C for 3 months), and skin permeation were determined by HPLC. The optimized micelles, prepared with Capryol 90^®^ and poloxamer, had particle sizes below 100 nm and exhibited superior skin permeation compared to oleanolic acid solutions in other surfactants. The formulation remained stable without phase separation or degradation during storage. In a clinical study with 23 female subjects, twice-daily application around the eyes for 8 weeks led to significant improvements in wrinkle-related skin parameters without irritation. These results demonstrate that oleanolic acid-loaded polymeric micelles are stable, safe, and effective for cosmetic anti-aging applications [163].

## 9. Clinical Relevance and Safety Profile of OA

### 9.1. Clinical Studies and Therapeutic Potential of OA

There are few clinical studies describing the effects of OA on the human body. One of the few available studies attempted to evaluate the hypolipidemic activity of OA in patients diagnosed with hyperlipidemia. Fifteen individuals were included in the observation, and OA was administered in tablet form for four weeks. The clinical status was assessed based on biochemical blood parameters collected before initiation and after completion of therapy. The results indicated a significant reduction in triglyceride and total cholesterol levels, as well as an improvement in the lipid profile. Microarray analysis revealed changes in the expression of 21 genes, including transcripts associated with lipid metabolism, insulin sensitivity and metabolic signaling [164].

A search of the clinicaltrials.gov database (accessed on 1 December 2025) using the intervention/treatment criterion “oleanolic acid” reveals five studies. A brief description of these studies is summarized in Table 2.

### 9.2. Toxicity and Safety Considerations

OA is characterized by relatively low acute toxicity; however, its adverse effects become apparent with long-term use or at high doses. OA may induce cholestatic-type hepatotoxicity. Studies on OA toxicity have been conducted mainly in mice, rats, and also in cell lines [170]. An experiment conducted on male C57BL/6J mice, which received oral OA administration for 4 days at two doses (457 mg/kg and 685.5 mg/kg), demonstrated that OA induces liver injury, as evidenced by increased ALT (alanine aminotransferase), AST (aspartate aminotransferase), ALP (alkaline phosphatase) and TBA (total bile acids) levels, histopathological changes, and disturbances in amino acid and energy metabolism—and most importantly—bile acid metabolism. The key mechanism of toxicity was found to be the accumulation of conjugated bile acids, indicating a cholestatic nature of the damage [171]. Study conducted on the Kunming mouse model showed that long-term administration of OA at low doses leads to hepatotoxicity, including increased ALT and ALP activity, hepatocyte degeneration, focal necrosis, and the development of fibrosis. This was accompanied by accumulation of the compound in plasma and liver, disturbances in bile acid metabolism, and activation of signaling pathways that promote oxidative stress and fibrosis progression [172]. In another study, orally administered OA at a dose of 100 mg/kg for 7 days did not induce adverse effects in healthy rats—no increases in ALT, AST or ALP were observed, and cholestasis parameters remained within normal ranges [173]. The determined oral LD_50_ value in both rats and mice exceeds 2 g/kg [174].

Available publications indicate that OA is safe at low concentrations corresponding to those found in food. Only long-term administration or high doses lead to the emergence of toxic effects, and the observed adverse outcomes do not represent acute toxicity.

## 10. Oleanolic Acid: Cytoprotection in Normal Cells vs. Pro-Apoptotic Effects in Cancer Cells

As was stated earlier in our paper, OA exhibits a context-dependent dual behavior, acting as an antioxidant and cytoprotective agent in healthy cells while displaying pro-oxidant and pro-apoptotic effects in cancer cells. In non-transformed cells, basal ROS levels are tightly regulated, and endogenous antioxidant systems remain intact. OA enhances cellular redox homeostasis primarily through indirect mechanisms, including activation of the Keap1-Nrf2-ARE pathway, which upregulates phase II detoxifying and antioxidant enzymes such as heme oxygenase-1, NAD(P)H: quinone oxidoreductase-1, superoxide dismutase, and glutathione-related enzymes, thereby mitigating oxidative stress and preserving mitochondrial integrity. In addition, OA suppresses pro-inflammatory signaling via NF-κB inhibition, further contributing to cytoprotection.

In contrast, cancer cells typically exhibit elevated basal ROS levels due to oncogenic signaling, mitochondrial dysfunction, and altered metabolism. Their antioxidant defenses are often near maximal capacity, making them vulnerable to further oxidative perturbations. OA treatment in malignant cells can exacerbate ROS accumulation, leading to oxidative damage, mitochondrial depolarization, and activation of intrinsic apoptotic pathways, including cytochrome c release and caspase activation. Simultaneously, OA inhibits survival pathways such as PI3K/AKT/mTOR and STAT3, while activating stress-responsive kinases including AMPK and MAPKs, promoting cell-cycle arrest and apoptosis.

Thus, the differential outcomes of OA in healthy versus cancer cells reflect context-dependent modulation of redox signaling. In normal cells, OA reinforces antioxidant defenses, restores redox balance, and supports survival, whereas in cancer cells it pushes ROS levels beyond the threshold of tolerance, inducing oxidative stress-mediated apoptosis. This selective redox modulation underlies OA’s potential as a chemopreventive and adjuvant anticancer agent, offering cytoprotection to normal tissues while targeting malignant cells.

## 11. Challenges and Limitations

Despite the promising pharmacological properties of oleanolic acid and its derivatives, challenges remain related to low aqueous solubility and bioavailability, which limits their clinical application. The low solubility of OA in aqueous environments requires the search for innovative formulation strategies that increase its bioavailability following oral or intravenous administration, which is crucial for achieving effective therapeutic concentrations in target tissues.

Furthermore, the complexity of oleanolic acid’s mechanisms of action, which involve modulation of multiple signaling pathways, requires further in-depth research to fully understand its therapeutic potential and optimize treatment protocols.

It is also essential to establish precise biomarkers of therapeutic response that will allow for monitoring treatment efficacy and early detection of potential resistance. Furthermore, it is necessary to thoroughly investigate the role of oleanolic acid in modulating the tumor microenvironment, including its effect on tumor-associated macrophages, which play a key role in tumor progression or regression. The development of formulation methods such as micellar systems, polymeric nanoparticles, or conjugates can significantly improve the pharmacokinetic properties of OA, increasing its solubility and bioavailability, and thus its in vivo effectiveness. Furthermore, analysis of potential interactions with chemotherapeutic drugs and radiotherapy is essential for developing a combined treatment strategy that could synergistically enhance therapy efficacy while limiting toxicity to healthy tissues. The low bioavailability of triterpenes, including oleanolic acid, is a well-documented problem resulting from their intensive first-pass metabolism in the liver, which requires the use of advanced chemical modifications or appropriate carriers.

Despite promising in vitro and in vivo results, there are no comprehensive, randomized phase II and III clinical trials confirming the efficacy and safety of oleanolic acid as a single anticancer agent or in combination therapy. This gap in clinical research represents a major limitation in translating laboratory findings into clinical practice, preventing widespread use of oleanolic acid in oncology treatment protocols.

## 12. Summary

This review systematizes current knowledge on oleanolic acid, highlighting its multifaceted effects in modulating oxidative stress and its therapeutic potential in carcinogenesis. A comprehensive analysis of its antioxidant mechanisms, including direct free radical scavenging and activation of endogenous defense systems, is presented, providing a basis for its use in cancer prevention and therapy. Additionally, its influence on key signaling pathways involved in cancer development and progression is discussed, suggesting its ability to modulate proliferation, apoptosis, and angiogenesis. The importance of chemical modifications of oleanolic acid, such as acetylation and esterification, to enhance its bioavailability and biological activity is also highlighted, opening new perspectives for the development of more potent derivatives. It is worth noting that pentacyclic triterpenes, such as oleanolic acid, represent a promising group of compounds in the search for new drugs, which is the subject of intensive preclinical research, although few have reached the pharmaceutical market to date. Among the few exceptions, omaveloxolone, an oleanolate derivative, was approved as the first effective drug for neurodegenerative disease, demonstrating the therapeutic potential of these compounds.

## 13. Conclusions

Oleanolic acid influences key signaling pathways involved in cancer initiation, promotion, and progression, offering promising prospects for the development of new therapeutic strategies. However, to fully exploit its potential, further in-depth mechanistic studies are necessary, particularly regarding interactions with key regulatory proteins and enzymes. It is also necessary to explore its potential in combination therapy, which could increase treatment efficacy while reducing the toxicity of conventional chemotherapeutics. Therefore, future research should focus on developing and testing nanocarriers for oleanolic acid, which could significantly improve its bioavailability and selectivity of delivery to cancer cells. Such approaches, for example, using nanoparticles that bind to tumor-specific ligands, could significantly increase therapy efficacy while minimizing adverse systemic effects.

On the other hand, research should focus on utilizing known and novel chemical modifications of the oleanolic acid molecule. These modifications should not only lead to the production of new derivatives with higher levels of cytotoxic activity against cancer cells and antioxidant activity, but also with a more favorable selectivity coefficient. Furthermore, the systemic toxicity of these compounds and their pharmacokinetic profile should be assessed in in vivo models to ensure the safety and effectiveness of their future clinical use. Detailed studies of the molecular mechanisms by which oleanolic acid and its derivatives modulate the cellular response to oxidative stress and influence carcinogenic pathways are essential to fully understand their therapeutic potential. Future work should particularly address the context-dependent modulation of Nrf2 and NF-κB by oleanolic acid, in order to exploit cytoprotective Nrf2 activation in normal tissues while driving oxidative stress and cell death in cancer cells.

Investigating the synergistic effects of oleanolic acid with other bioactive substances or drugs is also promising, as it could lead to the discovery of new, more effective anticancer strategies. These studies should include genetic and proteomic analyses to identify new molecular targets, as well as assess the effects of oleanolic acid on the tumor microenvironment and the host immune response. Finally, it is crucial to conduct rigorous clinical trials to confirm the efficacy and safety of oleanolic acid and its promising derivatives in the treatment of cancer patients, paving the way for their application in personalized medicine.

## Figures and Tables

**Table 2:** The specified criteria were used to search the clinical trials database (ClinicalTrials.gov).

Study Title	NCT Number	Interventions	Status	Ref.
Effect of Bile Acids on the Secretion of Satiation Peptides in Humans	NCT01674946	The intervention consists of intraluminal administration of TGR5 receptor (G-protein-coupled bile acid receptor) agonists—bile acids and OA—to stimulate GLP-1 (glucagon-like peptide 1) secretion and evaluate their effect on glucose metabolism in healthy volunteers. The control group receives saline perfusion, with an additional arm including OA supplementation.	Completed	[165]
Bioavailability of Oleanolic Acid Formulated as Functional Olive Oil	NCT05529953	The intervention involves administering to participants a functional olive oil enriched with A (30 mg OA in 55 mL olive oil) during breakfast and comparing it with a commercial control olive oil, with blood samples collected over 7 h to assess bioavailability and pharmacokinetic parameters. After a four-week washout period, participants undergo crossover administration of both types of olive oil.	Completed	[166]
Oleanolic Acid as Therapeutic Adjuvant for Type 2 Diabetes Mellitus	NCT06030544	The intervention involves daily consumption of 55 mL of olive oil—either enriched with OA or a commercial control olive oil—by participants with type 2 diabetes, divided across three main meals, for a period of 12 months. The olive oil intake will be combined with regular monitoring of metabolic, anthropometric, and biochemical parameters.	Active, not recruiting	[167]
Prevention With Oleanolic Acid of Insulin Resistance	NCT05049304	The intervention involves healthy adolescents consuming a meal containing either functional olive oil enriched with OA or regular olive oil, after which triglyceride-rich lipoprotein fractions will be isolated at 0, 2, and 5 h post-meal, and subsequently incubated with THP-1 macrophages (human monocytic leukemia cell line) to assess the anti-inflammatory activity of OA.	Completed	[168]
Six-Month Single-Blind, Placebo-Controlled Study of a Dietary Supplement on Hair Growth in 45 Volunteers	NCT06841458	The intervention involves daily oral administration of a supplement containing OA along with other plant-based ingredients (including pumpkin seed oil, *Saw Palmetto* extract, and L-cystine), aimed at promoting hair growth and reducing hair loss by inhibiting 5α-reductase activity. The control group receives a placebo.	Recruiting	[169]

## Data Availability

No new data were created or analyzed in this study. Data sharing is not applicable to this article.

## Abbreviations

The following abbreviations are used in this manuscript (abbreviations are listed in alphabetical order):

^●^OH	hydroxyl radical
^1^O_2_	singlet oxygen
2008	human epithelial ovarian carcinoma cell line
2008.C-13	human epithelial ovarian carcinoma cell line
2774	human epithelial ovarian carcinoma cell line
3-MA	3-methyladenine
5-FU	5-fluorouracil
β-TrCP	beta-transducin repeat-containing protein
μg/mL	microgram per milliliter = 10^−6^ g per 10^−3^ L
μM	micromole = 10^−6^ moles
A-549	adenocarcinomic human alveolar basal epithelial cell line
ADMETox	absorption, distribution, metabolism, excretion and toxicity
AGS	gastric cancer cell line
AKR1B10	aldo-keto reductase family 1 member B10
AKT	protein kinase B, known as PKB
AKT1	AKT serine/threonine kinase 1
AKT/JNK	two distinct but interconnected cell signaling pathways that play roles in cell survival, apoptosis, and other cellular processes
AKT/mTOR/S6K	crucial molecular pathway that regulates cell growth, proliferation, protein synthesis, and metabolism
AKT/PKB	protein kinase B
ALT	alanine aminotransferase
AMPK	AMP-activated protein kinase
AP-1	activator protein 1
ASK-1	apoptosis signal-regulating kinase 1
ASP-831	aspartic acid located at position 831 in the protein
AST	aspartate aminotransferase
ATP	adenosine triphosphate
B-16	murine melanoma cell line
B-16-F-10	murine melanoma cell line
BALB/c	albino, laboratory-bred strain of the house mouse
BAX/BCL-2	a relationship between BCL-2-associated X protein and B-cell lymphoma 2 protein
BCL-2	B-cell lymphoma 2 protein
BCL-xL	anti-apoptotic protein that promotes cell survival by preventing programmed cell death, or apoptosis
BEAS-2B	human lung epithelial cell line
Beclin1	protein that in humans is encoded by the BECN1 gene
BEL-7402	human hepatocellular carcinoma cell line
BER	base excision repair system
bFGF	basic fibroblast growth factor
BGC-823	human gastric cancer cell line
BIM	BCL-2–interacting mediator of cell death
C-3, C-12, etc.	number of C atom within the molecule of a compound (in our publication: within molecule of OA)
CAOV-3	human epithelial ovarian carcinoma cell line
CBP/p300	CREB-binding protein (CBP) and p300 (or EP300), transcriptional coactivators
CDDO	2-cyano-3,12-dioxooleana-1,9(11)-dien-28-oic acid
CDDO-Me	2-cyano-3,12-dioxooleana-1,9(11)-dien-28-oic acid methyl ester
CDKs	cyclin dependent kinases
CDDO-Me	2-cyano-3,12-dioxooleana-1,9(11)-dien-28-oate
CEM	human T-cell leukemia cell line
cm/s	centimeter per second
cryo-SEM	cryo-scanning electron microscopy
CSCs	cancer stem cells
DISC	death-inducing signaling complex
DNA	deoxyribonucleic acid
DPPH	2,2-diphenyl-1-picrylhydrazyl
DR4/DR5	death receptor 4 and death receptor 5
DSC	differential scanning calorimetry
DU-145	human prostate cancer cell line
EGFR	epidermal growth factor receptor
EMT	epithelial-to-mesenchymal transition
EPR	enhanced permeability and retention
ER	endoplasmic reticulum
ERK	extracellular signal-regulated kinase, mainly ERK1 and ERK2
ERK1/2	extracellular signal-regulated kinases 1 and 2
F%	bioavailability
FTIR	Fourier-transform infrared
g/mol	grams per mole, a unit of molar mass
G-361	human melanoma cell line
GLI-1	glioma-associated oncogene homolog 1
GLP-1	glucagon-like peptide 1
GMC	ganglion mother cells
GPx	glutathione peroxidase
GSK3β	glycogen synthase kinase 3 beta
H-22	human hepatocellular carcinoma cell line
H_2_O_2_	hydrogen peroxide
HCT-116	human colorectal carcinoma 116
HDAC3	histone deacetylase 3
HDF	human dermal fibroblasts
HeLa	human epithelial adenocarcinoma cell line
Hep-3B	human hepatocellular carcinoma cell line
Hep-G2	human hepatocellular carcinoma cell line
HEY	human epithelial ovarian carcinoma cell line
HL-60	human leukemia cell line
HL-7702	normal human liver cell line
HNO_2_	nitric acid
HO-1	heme oxygenase-1
HPLC	high-performance liquid chromatography
HPLC-MS	high-performance liquid chromatography–mass spectrometry
HR	homologous recombination repair system
HT-29	human colorectal adenocarcinoma cell line
Huh-7	human hepatocellular carcinoma cell line
HUVEC	human umbilical vein endothelial cell line
IκBα	inhibitor of kappa B alpha
IC_50_	half maximal inhibitory concentration
IL-1β	interleukin-1 beta
IL-6	interleukin-6
IMR-32	human neuroblastoma cell line
IUPAC	The International Union of Pure and Applied Chemistry
JAK2	Janus kinase 2
JAK2/STAT3	crucial intracellular signaling cascade that regulates cell growth, differentiation, and immune function in response to extracellular signals like cytokines and growth factors
JNK	c-Jun N-terminal kinase
KB	human epidermal oral carcinoma cell line
Ki-68	proliferation marker
KRAS	Kirsten rat sarcoma virus
L-1210	murine lymphocytic leukemia cell line
L02	normal human hepatic cell line
LC3-I, LC3-II	microtubule-associated protein 1 or protein 2 light chain 3
LC3-II/LC3-I	ratio of two forms of the autophagy-related protein LC3, i.e., microtubule-associated protein 1A/1B-light chain 3
LCB-II	lipidated form of the protein LC3B = microtubule-associated protein 1 light chain 3 Beta
LNCaP	human prostate cancer cell line
LPS	lipopolysaccharides
LY294002	2-(4-morpholinyl)-8-phenyl-4H-1-benzopyran-4-one
LYS 721	lysine located at position 721 in the protein
MAFK	v-maf avian musculoaponeurotic fibrosarcoma oncogene homolog K
MAPK	mitogen-activated protein kinase
MCF-10A	non-tumorigenic human mammary epithelial cell line
MCF-12A	normal breast epithelial cell line
MCF-7	human breast cancer cell line
MCTs	medium-chain triglycerides
MDA-MB-231	human metastatic breast cancer cell line
MDA-MB-435	human metastatic breast cancer cell line
MDM2	mouse double minute 2 homolog
mg/kg	milligrams per kilogram = 10^−3^ g per 10^3^ g
mg/L	milligrams per liter = 10^−3^ g per liter
mg/mL	milligrams per milliliter = 10^−3^ g per 10^−3^ L
MKN-28	gastric cancer cell line
MM-GBSA	molecular mechanics/generalized born surface area
MMP-7	invasion marker
MMPs	matrix metalloproteinases
mPa x s	millipascal-second = 10^−3^ pascals-second
mPEG-P(D,L)LGA	methoxy poly(ethylene) glycol and poly(D,L-lactic-*co*-glycolic acid) poliester
mPGE-PLA	methoxy poly(ethylene) glycol and poly(lactic acid) poliester
mPEG-PLGA	methoxy poly(ethylene) glycol and poly(lactic-*co*-glycolic acid) poliester
mTOR	mechanistic target of rapamycin or mammalian target of rapamycin
MTT	3-(4,5-dimethylthiazol-2-yl)-2,5-diphenyltetrazolium bromide
NADPH	nicotinamide adenine dinucleotide phosphate
Nanog, CD-133, Oct-4, SOX2	transcription factors which are stemness genes
NCI	The National Cancer Institute
NEm	nanoemulsion
NER	nucleotide excision repair system
NF-κB	nuclear factor kappa B cells
NHEJ	non-homologous end joining repair system
nm	nanometer = 10^−9^ m
NMP-1	human epithelial ovarian carcinoma cell line
NO	nitric oxide
NO_2_	nitrogen dioxide
NQO1	NAD(P)H quinone oxidoreductase 1
Nrf2	nuclear factor erythroid 2-related factor 2
^•^OH	hydroxyl radical
O_2_^−^	superoxide anion
OA	oleanolic acid
OAnano	nanoformulation of oleanolic acid
OA-MVLs	oleanolic acid-encapsulated multivesicular liposomes
OH^−^	hydroxide anion
OSCC	oral squamous cell carcinoma cell line
OVCAR-3	human epithelial ovarian carcinoma cell line
o/w	oil and water
P/D	phospholipid-to-drug ratio
P21, p27	cell cycle regulators
p38	group of mitogen-activated protein kinases
p53	tumor protein p53
p65	subunit of the NF-κB transcription factor
Panc1	pancreatic carcinoma-1 cell line
P_app_	apparent permeability coefficient
PARP	poly-(ADP-ribose) polymerase
PC-3	human prostate cancer cell line
PEG	polyethylene glycol
PGE_2_	prostaglandin E_2_
pGI_50_	negative logarithm of the half-maximal inhibitory concentration
pH	negative logarithm of the concentration of hydrogen ions (H^+^) in a solution
PI3K	phosphatidylinositol 3-kinase
PI3K/AKT	crucial intracellular signaling cascade that regulates cell survival, growth, and proliferation
PI3K/AKT/mTOR	crucial intracellular signaling pathway that regulates cell growth, proliferation, survival, and metabolism
pLC_50_	negative logarithm of the half-maximal lethal concentration
pTGI	negative logarithm of the total inhibition of cell growth
PVP	polyvinylpyrrolidone
RAW 264.7	macrophage-like, Abelson leukemia virus-transformed cell line
RNS	reactive nitrogen species
ROR	Research Organisation Registry
ROS	reactive oxygen species
ROS/RNS	reactive oxygen species and reactive nitrogen species
SAR	structure–activity relationship
SD	solid dispersion
SHH	sonic hedgehog gene
SI	selectivity index
SiHa	human cervical squamous cell carcinoma cell line
SKOV-3	human epithelial ovarian carcinoma cell line
SMEDDS	self-microemulsifying drug delivery system
SMMC-7721	human hepatocellular carcinoma
Snail, Slug, Twist	transcription factors which are EMT genes
SOD	superoxide dismutase
STAT3	signal transducer and activator of transcription 3
SW-480	human colon adenocarcinoma
t_1/2_	half-release time
T-24	human bladder cancer cells
TGF-β1	tumor growth factor β1
TGF-β1/Smad3/Snail	molecular signaling cascade where TGF-β1 (Transforming Growth Factor-beta 1) initiates a series of events involving Smad3 and Snail to drive cellular processes like epithelial–mesenchymal transition (EMT)
TGR5	G-protein-coupled bile acid receptor
THP-1	human monocytic leukemia cell line
TNF-α	tumor necrosis factor α
TRAIL	TNF-related apoptosis-inducing ligand
U-14	human cervical carcinoma cell line
U-87	human glioblastoma cell line
U-937	monocytic human cell line derived from histiocytic lymphoma
UA	ursolic acid
ULK1	serine/threonine kinase that plays a central, initiating role in autophagy
UV	ultraviolet
var.	varietas, which means variety
VEGF-A	vascular endothelial growth factor A
Wnt, Notch, EGFR	types of cell signaling pathways that are crucial for processes like embryonic development, cell proliferation, differentiation, and migration
